# Phylogenomics Reveals Three Sources of Adaptive Variation during a Rapid Radiation

**DOI:** 10.1371/journal.pbio.1002379

**Published:** 2016-02-12

**Authors:** James B. Pease, David C. Haak, Matthew W. Hahn, Leonie C. Moyle

**Affiliations:** 1 Department of Biology, Indiana University, Bloomington, Indiana, United States of America; 2 Department of Plant Pathology, Physiology and Weed Science, Virginia Tech, Blacksburg, Virginia, United States of America; 3 School of Informatics and Computing, Indiana University, Bloomington, Indiana, United States of America; Massey University, NEW ZEALAND

## Abstract

Speciation events often occur in rapid bursts of diversification, but the ecological and genetic factors that promote these radiations are still much debated. Using whole transcriptomes from all 13 species in the ecologically and reproductively diverse wild tomato clade (*Solanum* sect. *Lycopersicon*), we infer the species phylogeny and patterns of genetic diversity in this group. Despite widespread phylogenetic discordance due to the sorting of ancestral variation, we date the origin of this radiation to approximately 2.5 million years ago and find evidence for at least three sources of adaptive genetic variation that fuel diversification. First, we detect introgression both historically between early-branching lineages and recently between individual populations, at specific loci whose functions indicate likely adaptive benefits. Second, we find evidence of lineage-specific de novo evolution for many genes, including loci involved in the production of red fruit color. Finally, using a “PhyloGWAS” approach, we detect environment-specific sorting of ancestral variation among populations that come from different species but share common environmental conditions. Estimated across the whole clade, small but substantial and approximately equal fractions of the euchromatic portion of the genome are inferred to contribute to each of these three sources of adaptive genetic variation. These results indicate that multiple genetic sources can promote rapid diversification and speciation in response to new ecological opportunity, in agreement with our emerging phylogenomic understanding of the complexity of both ancient and recent species radiations.

## Abstract 2

Wild tomatoes contain immense natural trait diversity; this study describes the evolutionary processes that have generated this diversity over only a few million years, drawing from multiple sources of genetic variation.

## Author Summary

The formation of new and distinct species during evolution often occurs in rapid bursts of diversification in which many species arise within a short time frame. The ecological and genetic factors that promote these radiations are much debated. Here, we examine genome-wide patterns of molecular evolution that accompanied a rapid adaptive radiation among 13 species of wild tomato—the ecologically and reproductively diverse group that gave rise to the domesticated tomato. By analyzing patterns of genetic variation in thousands of expressed genes from multiple populations and species, we identify genome-wide signatures of rapid consecutive speciation events during 2.5 million years of diversification in this group. These signatures include pervasive shared ancestral variation and frequently discordant signals of relatedness among different parts of the genome. Our analyses find evidence for three unique sources of genetic variation that fuel adaptive diversification in this group—postspeciation hybridization, rapid accumulation of new mutations, and recruitment from ancestral variation—and identify specific examples of putatively adaptive loci drawn from each source. Recent analyses of other rapid radiations have also inferred a role for at least one of these mechanisms; our finding of all three simultaneously at work within the same diversifying clade suggests that they might be a universal feature of rapid adaptation to diverse environmental niches.

## Introduction

Speciation—the origin of new species—occurs when diverging lineages accumulate ecological, functional, and/or reproductive differences that result in their evolutionary independence from close relatives. Rates of speciation vary widely among groups, but the underlying causes of this rate variation, especially the conditions that promote bursts of adaptive divergence across short timescales (“adaptive radiations”), are still under debate [1–6]. New ecological opportunity, although likely essential, appears to be insufficient as the sole explanation for many contemporary cases of species radiation [7,8]. Instead, intrinsic factors might be more critical, including the availability of sufficient genetic variation to respond to ecological conditions or of novel traits that accelerate rates of diversification [2,9]. To understand these factors requires a detailed understanding of both the ecological transitions and the underlying molecular genetic changes that accompany, and potentially facilitate, speciation.

Whereas classical genetic studies of speciation were often limited to relatively few loci or genomic regions, modern sequencing can interrogate genome-wide patterns of molecular differentiation during speciation and can potentially reveal the genetic substrate of associated trait changes. Recent studies have begun to uncover several intriguing patterns of phylogenomic divergence, especially in rapidly radiating groups. One such pattern is a persistent discordance among genes for particular phylogenetic relationships, regardless of the quantity or quality of molecular data sampled. This discordance is often caused by incomplete lineage sorting (ILS), in which shared ancestral variation fails to fix between closely timed speciation events [10,11]. This sorting of ancestral variation causes conflicting phylogenetic signals, and is especially common in groups radiating both in recent history (e.g., African rift cichlids [7], *Drosophila simulans* group [12], platyfish [13], and horses [14]) and at deeper timescales (major land plant families [15] and major bird lineages [16]). A second emerging pattern is that postspeciation hybridization (introgression) appears to be substantially more commonplace than previously appreciated. A diverse range of animal groups—including butterflies, horses, fish, flies, mosquitoes, and Galápagos finches [8,12–14,17,18]—all show evidence of postspeciation gene flow. The frequency and extent of introgression is remarkable given that introgressive hybridization has played little role in conventional models of animal speciation and diversification [19].

Overall, both ILS and postspeciation introgression contribute to generating more complex evolutionary histories than can be represented by simple bifurcating trees. In response, new approaches are being developed to account for these potential sources of gene tree discordance, including tools that can infer the underlying species tree even when there are high levels of ILS (e.g., MP-EST [20], ASTRAL [21]). Accordingly, despite these complexities, several genome-wide studies of diversification have successfully clarified ambiguous species relationships and highlighted loci that might underpin specific functional or ecological changes accompanying rapid phylogenetic transitions. These include loci contributing to ecologically and reproductively significant traits, such as beak size differentiation among Galápagos finches [18].

**Fig 1:**
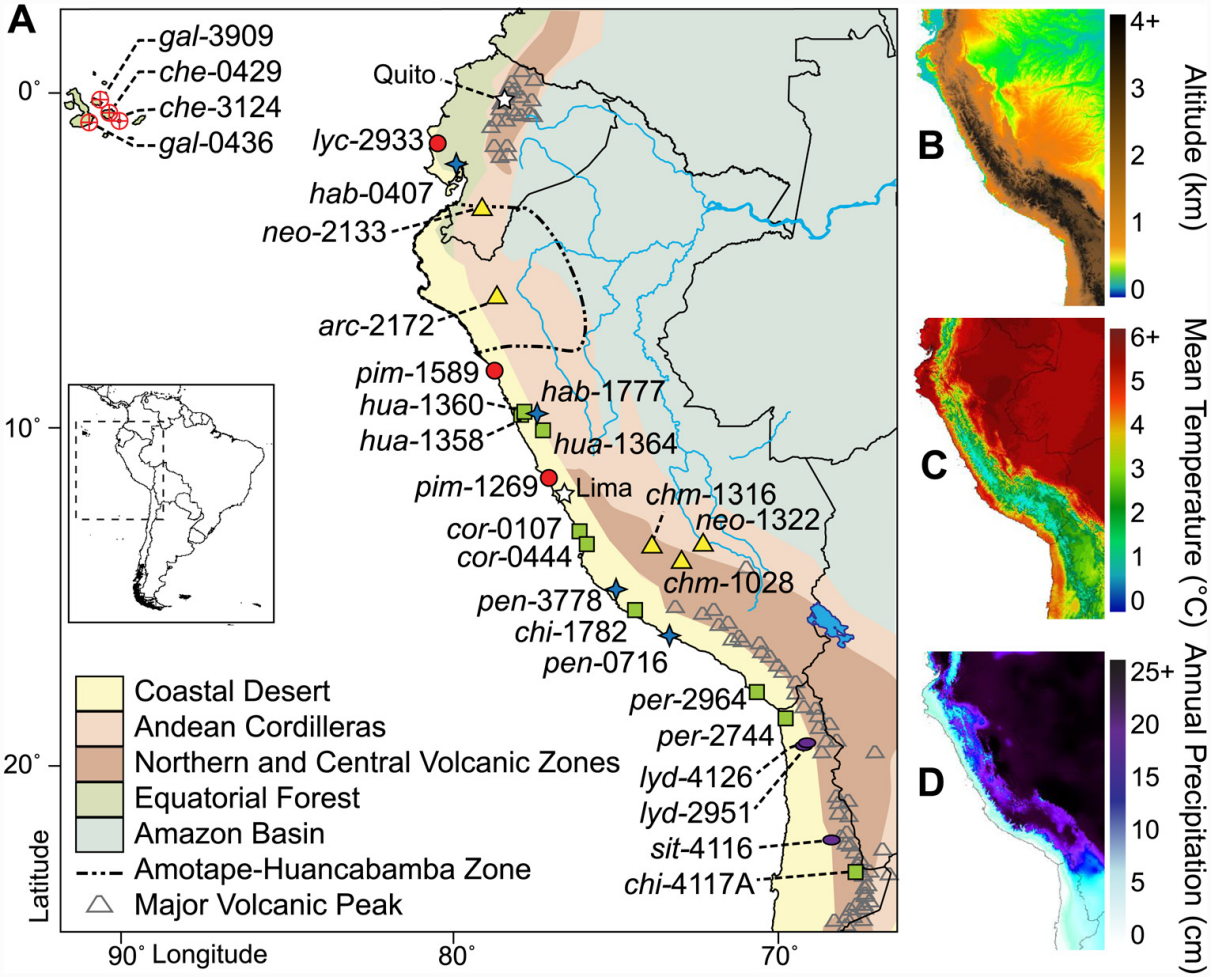
Geographic distribution and ecological diversity of sampled populations of wild tomato. (A) Wild tomato species inhabit diverse ecological zones (shaded regions) along the western coast of South America and the Galápagos Islands. For each sample location, labels indicate species and accession number, and symbols denote major phylogenetic groupings (circle = Esculentum, triangle = Arcanum, square = Peruvianum, star = Hirsutum, oval = outgroup; base map modified from original from http://www.freevectormaps.com). High variation of (B) altitude, (C) mean annual temperature (D), and annual precipitation across the habitat range of wild tomato species (data from http://www.worldclim.org; plotted using GRASS GIS http://grass.osgeo.org/).

Here, we examine genome-wide patterns of lineage divergence among all species in the wild tomato clade (*Solanum* sect. *Lycopersicon*) using whole transcriptome sequencing (RNA-Seq). In addition to domesticated tomato (*S*. *lycopersicum*) and its conspecific wild relative, the group includes 12 species native to the Galápagos Islands and Andean South America, a biodiversity hotspot (Fig 1 and S1 Fig), and the clade has been estimated to share a common ancestor ~2 million years ago (Ma) [22]. All lineages are diploid and chromosomally homosequential, except for a few small rearrangements that distinguish some species [23–25]. Wild tomato species are differentiated for numerous functional, ecological, and reproductive traits, and display different habitat associations at macroecological scales (Fig 1) [26–29]. They also exhibit strong, but often incomplete, reproductive isolating barriers at various pre- and postzygotic stages [30–35]. Nonetheless, efforts to infer species phylogenies and the timing of lineage divergence have met with mixed success and have revealed chronically challenging taxa in this group [23,27,36,37].

Given its ecological and reproductive diversity, the wild tomato group presents a unique opportunity to examine the genome-wide signatures of rapid recent divergence. While the timescale of speciation is comparable to other recent phylogenomic studies of radiating animal clades (e.g., [7,17,18,38]), it is unclear whether plant clades such as wild tomatoes—equally rapidly radiating, but also classically perceived as having greater tendency to hybridize [39]—will differ in their genomic patterns of diversification and introgression and in the genetic variation on which this diversification is based. The aims of our study were, first, to clarify species phylogenetic relationships; second, to assess postspeciation gene flow between lineages; and third, to investigate the genomic basis of lineage-specific and environment-specific adaptation. We find evidence consistent with at least three genetic sources of adaptive variation: introgression among species, de novo mutation, and recruitment from ancestral variation. Our results indicate that a combination of all three of these evolutionary factors facilitated rapid adaptive expansion in response to ecological opportunity.

## Results and Discussion

### Phylogenomic Discordance Is due to Extensive ILS Caused by Rapid Diversification

We sequenced whole transcriptomes (mRNAs) for 29 accessions from 13 tomato species and 2 outgroup species (Fig 1 and S1 Table). Although these sequences came from different species, high sequence similarity allowed us to confidently align ~90% of RNA-Seq read-pairs from all accessions to the reference genome of the domesticated tomato, *S*. *lycopersicum* [40]. We aligned an average of 31.6 Mb per accession, covering 21,896 genes with an average of >26 accessions per gene. This corresponds to an average coverage of 76% of total annotated coding regions per accession, but only 3.9% of the full genome due to the high proportion of gene-poor heterochromatin in the tomato genome [40,41].

**Fig 2:**
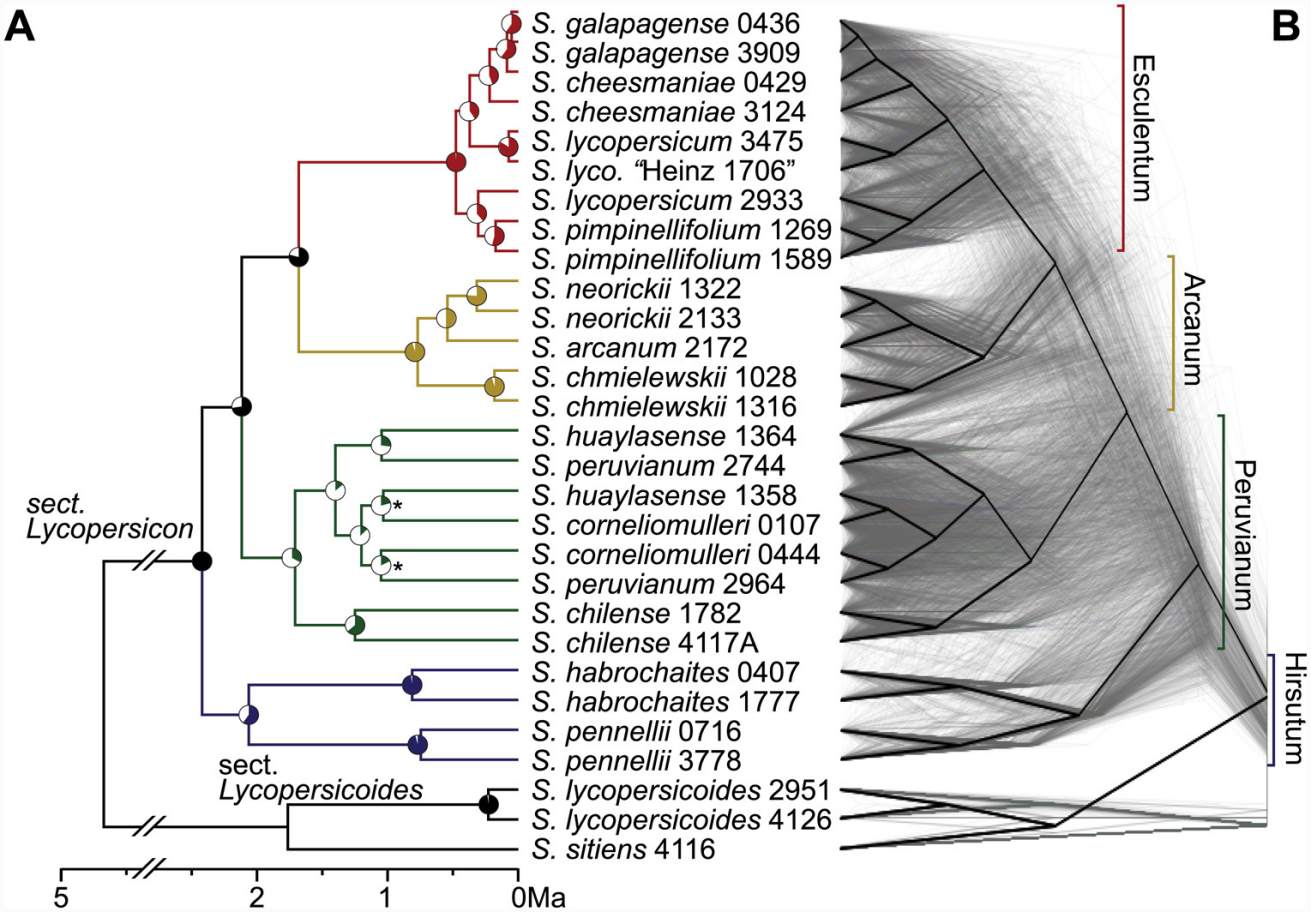
The phylogeny of *Solanum* sect. *Lycopersicon*. (A) A whole-transcriptome concatenated molecular clock phylogeny with section *Lycopersicoides* as the outgroup. Branch colors indicate the four major subgroups (labels on right). Pie charts on each node indicate majority rule extended bipartition support scores (out of 100) using trees from 100-kb genomic windows. All nodes are supported by 100 bootstrap replicates, except “*” denotes bootstrap support score of 68. (B) A “cloudogram” of 2,745 trees (grey) inferred from nonoverlapping 100-kb genomic windows (see also S2 Table). For contrast, the consensus phylogeny is shown in black.

We inferred phylogenetic relationships among species using several data partitions, including: whole transcriptome concatenated, each chromosome concatenated, nonoverlapping 1 Mb and 100 kb genomic windows, and trees inferred from individual genes (Fig 2, S2 Fig, and S2 Table). We also used a majority rule method (as implemented in RAxML [42]), a coalescent method (MP-EST [20]), and a coalescent-based quartet method (ASTRAL [21]) to infer phylogenies using the 100-kb window trees (S2D–S2F Fig). All concatenation, majority rule, and coalescent methods inferred a generally consistent species tree topology (Fig 2A), identifying four main groups that recapitulate relationships found in previous studies [23,27,36,37]. As in other recent analyses, we find that *S*. *habrochaites* and *S*. *pennellii* are placed together (the “Hirsutum” group) and split from the other wild tomatoes at the base of the tree [37], that *S*. *arcanum* groups with other members of our inferred “Arcanum” group rather than with the “Peruvianum” group [43], and that some members of the “Peruvianum” group have ambiguous taxonomic placement, especially accessions of *S*. *huaylesense* [37]. In particular, one of our lineages of *S*. *huaylesense* shows evidence of extensive and recent reticulation (as discussed further below), so it was omitted from our reconstructed consensus tree (Fig 2A). Using molecular clock estimates, we dated several nodes that define major groups and distinct species. Our inferred date for the basal node (2.48 Ma) agrees well with a recent fossil-calibrated estimate of 2 Ma [22], and we confirm that some groups within the clade have very recent divergence times (e.g., <0.5 Ma for the Esculentum or “red-fruited” group; Fig 2).

Because of the large amount of sequence used in the concatenated whole transcriptome alignment (46.5 Mb with at least 10 accessions represented) and the large number of loci used in coalescent-based methods (*n* = 2,745 100-kb windows), our phylogeny shows strong bootstrap support for almost all nodes (Fig 2A), as expected [44]. However, these summary support measures conceal rampant phylogenetic complexity that is evident when examining the evolutionary history of more defined genomic partitions (Fig 2B, S2 Fig, and S2 Table). Among the 2,745 trees generated from nonoverlapping 100 kb segments of the genome, we inferred 2,743 different topologies and found wide variation in support for the specific placement of individual accessions and species (S2 Table). For example, the Esculentum group is supported by ~99% of 100-kb trees, while the more diffuse Peruvianum group is supported by only 21.3% of trees. Gene trees show discordance both within subclades and across deeper nodes and, when examined spatially within the genome (using “chromoplots” [17,45]), discordant topologies are observed to be interdigitated across all chromosomes (S2, S4 and S5 Figs). None of the trees generated from 100-kb segments (Fig 2B) matched the topology of the species tree (Fig 2A). We find that shorter internodes exhibit more discordance (S3C Fig), indicating that homoplasy can be excluded as major contributor to the observed discordance [46], but consistent with high levels of ILS due to rapid speciation in the group (S1 Text Section 3.1). As such, our results are clearly concordant with several other recent studies of contemporary (e.g., [7,14,18,47]) and more ancient (e.g., [15,16,46,48]) adaptive radiations that also detect abundant evidence for genome-wide ILS.

### Ancestral Polymorphism Is Broadly Shared among Present Subclades

**Fig 3:**
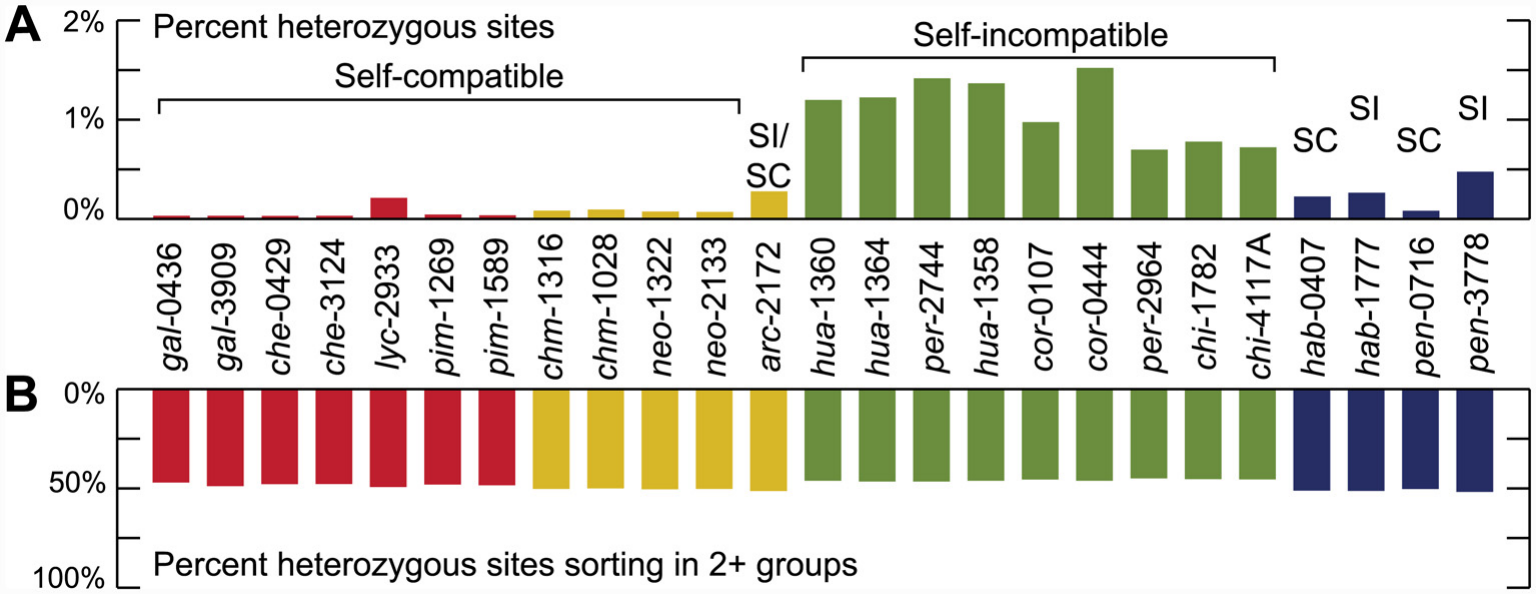
Low variation and pervasive sorting of ancestral heterozygosity in the wild tomato clade. (A) The fraction of heterozygous sites within accessions and (B) the proportion of heterozygous sites in each accession that are sorting in other species.

To investigate these patterns of discordance further and to more accurately assess heterozygosity in these wild species, we used a high-depth (HD) dataset of 12.1 million sites with ≥10X sequencing coverage for all samples. Consistent with very recent divergence, tomato species differ on average by ~1% nucleotide divergence, ranging from 0.05% between Galápagos species to 1.58% between the most distantly related pairs (full table in S1 Data 1.2). Within-accession variation ranged from 0.05%−1.1% heterozygous sites (Fig 3A) and was higher in outcrossing (self-incompatible) lineages compared to more inbreeding (self-compatible) lineages, as expected [49,50]. In contrast, the proportion of loci that showed shared genetic variation across major subclades was approximately the same across all accessions (Fig 3B); that is, all lineages appear to exhibit equivalent levels of shared ancestral genetic variation, regardless of their overall proportion of heterozygous sites.

### Introgression after Speciation Varies Widely among Lineages

Since both ILS and introgression manifest as discordant phylogenetic relationships, distinguishing these two factors is challenging, even with new methods developed specifically to address this issue [51,52]. Nonetheless, we detected evidence for a highly variable history of cross-species introgression, including one clearly reticulate lineage, a few cases of clearly demarcated and chromosomally localized introgressions between lineages, and many lineages with little or no evidence of introgression (S4 and S5 Figs). These observations in wild accessions are in addition to observed evidence of intentional introgression of wild alleles into domesticated accessions, which are presumably for crop improvement [53] and well documented in other studies [54,55] but excluded here to focus on introgression in nature.

In the case of reticulate lineages, *hua*-1360 (in particular), and *hua*-1364 and *per*-2744 (to a lesser extent) show extensive phylogenetic conflict and patterns of recent hybridization (S4 Fig). In *hua*-1360, 48% of gene trees indicate that this lineage has a closer relationship with the Esculentum group than the Peruvianum group, where it has traditionally been placed based on morphological and reproductive characters [56]. Our finding agrees with another recent study that found this accession to be admixed [43], and with our analysis indicating that, of all accessions analyzed here, this lineage has the highest taxonomic instability index [57], a metric of the consistency of topological placement of individual taxa in a phylogeny (S1 Data 1.26). In addition, 40% of heterozygous sites in *hua*-1360 contain at least one allele that is otherwise Peruvianum- or Esculentum-specific (S4 Fig), indicating that the hybridization event that produced this accession is relatively recent. Unsurprisingly, including this reticulate lineage when inferring the whole-transcriptome phylogeny causes the Peruvianum group to appear to be paraphyletic with respect to the Esculentum and Arcanum groups (S2A Fig). While the level of reticulation observed in these three lineages was surprisingly high, it is consistent with both the history of contested species definitions in the Peruvianum group and the particularly uncertain status of *S*. *huaylasense*—a recently described species with populations that have had conflicting taxonomic designations [43,58].

To assess introgression across all species in the clade, we calculated genome-wide *D*-statistics [51,59]. In addition, for nonoverlapping genomic windows, we computed *D*-statistics and *D*_FOIL_ statistics [52] to identify spatially localized regions of introgression. Because there are 2,925 trios of taxa that can be analyzed in the *D-*statistic framework, we inferred the timing of introgression based on shared signals among related species. Based on genome-wide *D-*statistics, we inferred that the majority of introgression occurred among relatively ancient lineages rather than across more recent splits (S5 Fig and S1 Text Sections 1.5 and 4.2). To estimate the proportion of the euchromatic fraction of the genome exchanged in these ancient events, we calculated the frequencies of discordant gene trees for trios of accessions, using one representative from each lineage that was implicated in introgression by the *D*-statistics. Other than the reticulate genomes previously described, we noted two likely ancient introgression events (S1 Text Section 4.2 and S4E and S4F Fig). First, extrapolating from the frequency of windows with significant *D*-statistics observed in our transcriptome data, the ancestor of *S*. *habrochaites* is inferred to have exchanged 8.7% of the euchromatic portion of its genome with the lineage that gave rise to the Esculentum and Arcanum groups. The other ancient introgression involved an estimated 8.8% genome exchange between the lineages ancestral to the Esculentum+Arcanum groups and the Peruvianum group, though these patterns are more difficult to interpret because of both ancestral population structure and interbreeding among Peruvianum group species (S4 and S5 Figs).

**Fig 4:**
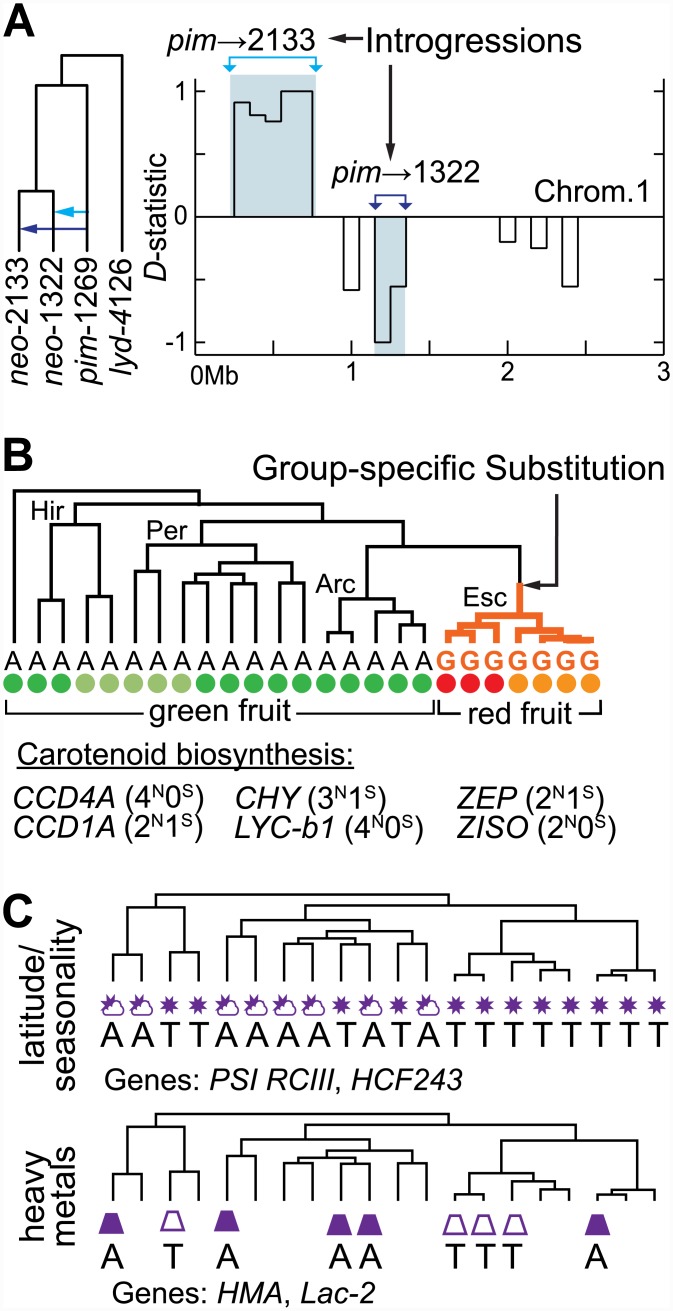
Multiple modes of molecular adaptation. (A) The *D*-statistic for testing introgression for 100-kb windows on the short arm of chromosome 1 using the tree shown. Shaded regions indicate windows where introgression is significantly detected (*p* ≤ 1.45 × 10^−4^; see also S1 Data 1.5 and S5A Fig). (B) Alleles are shown indicating an example of a lineage-specific substitution for the red-fruited Esculentum group, with colored dots indicating approximate fruit colors for each accession. Genes directly involved in the carotenoid biosynthesis pathway were found to have several nonsynonymous (^N^) and synonymous (^S^) substitutions specific to the red-fruited lineage. (C) Environment-specific alleles found in groups of accessions from different phylogenetic groups but under common environmental stressors for consistent (sun) or variable (sun/cloud) climate (top) or the presence (filled symbol) or absence (empty symbol) of heavy metals in the environment (bottom).

Except in the case of very recent introgression between several Peruvianum group accessions (S5E Fig), evidence of more recent introgression between species or accessions is limited to a few cases and involves <1% of our analyzed loci (Fig 4A, S5 Fig, and S1 Text Section 4.2). In particular, each of the two *S*. *neorickii* accessions has a different region introgressed from a red-fruited clade donor (Fig 4A). Another case involves introgression from the red-fruited clade into only one *S*. *pennellii* accession (S1 Text Section 4.2 and S5D Fig). These cases are particularly interesting because of their recent timing, since population-specific introgressions must postdate the common species ancestor. Based on the function of the genes involved, they may also represent strong candidates for adaptive introgression [60–62]. For example, the two independent introgressions into *S*. *neorickii* correspond to different regions within the *Cf*-4/*NL* (“Northern Lights”) locus that is associated with resistance to the pathogenic leaf mold *Cladosporium fulvum* [63,64]. Because these two accessions of *S*. *neorickii* were sampled from ecologically distinct habitats ~1,350 km apart, it is plausible that the introgressions occurred in response to different local fungal pathogens. In contrast, the introgression into *S*. *pennellii* involved transfer of a gene currently without a described environment-specific adaptive role (*Solyc08g005190*; *pre-mRNA-splicing factor cwc22*).

Finally, looking across all branches and all possible trios of species within the wild tomatoes, we can infer a coarse clade-wide estimate of the frequency with which introgression appears in our dataset, if we assume an arbitrary but reasonable general cutoff for inferring significant evidence of introgression. For example, across all 26 lineages that we queried within the wild tomato tree, we found 1,147 windows where |*D*| ≥ 0.2, *p* < 1 × 10^−4^, and |ABBA − BABA| ≥ 10 for any trio of three species (out of 2,596 100-kb windows with 100 or more aligned sites). That is, about 44% of windows show some evidence of introgression over at least one branch in the tree, as expected, given that our overall sampling of taxa was found to include admixed taxa. On the basis of these criteria, then, per branch we find that 1.76% of our 100-kb windows show evidence of introgression. Note that if we remove the substantially admixed taxa (*hua*-1360, *hua*-1364, and *per*-2744) from these calculations, we find 672 windows that are significant over the 20 remaining possible branches, and therefore that an estimated 1.29% (672 / [(2,596)(20)]) of windows show evidence of introgression. These calculations rely on several simplifying criteria, but they permit a crude estimate of the genome-wide proportion of 100-kb windows that show evidence of past introgression, and therefore that could contribute to adaptive allele sharing between lineages. Nonetheless, it is clear that a major computational need for future phylogenomic studies is a method to simultaneously integrate data from more than four taxa in order to infer the number and specific timing of introgression events among all members of a clade. Regardless, the substantial but small estimate of clade-wide introgression we infer here also suggests that the pervasive genome-wide discordance we detect across the clade is predominantly due to the effects of ILS.

### De Novo Evolution of Lineage-Specific Ecological Traits

Despite the extensive phylogenetic complexity observed in our genome-wide data, wild tomato species and subclades are separated by clear diagnostic ecological preferences, functional traits, and various pre- and postzygotic isolating barriers [26,27]. Therefore, in addition to shared ancestral variation and introgressed alleles, there should also be mutations that uniquely diagnose well-supported groups within the clade, including in loci that confer species- and group-specific traits. To identify candidates for such loci, we examined patterns of protein-coding changes to distinguish genes that showed high rates of group-specific protein-coding changes relative to group-specific synonymous changes. Because the high level of ILS detected here, in addition to lineage-specific introgression, produces highly discordant gene trees, standard approaches for inferring the timing of nucleotide substitutions may be inaccurate [65]. Therefore, we used a more conservative *d*_N_/*d*_S_-like test to identify genes with high numbers of unambiguously clade-specific sequence changes. This test requires that an amino acid substitution be exclusively observed in a particular group and be common to all members of the group (S1 Text Section 5).

For each of the four main groups within wild tomatoes (Fig 2B), we found hundreds to thousands of genes with protein-coding changes that were unique to all species within a group and not found in other groups (including the outgroup) (S1 Data 1.8−1.20). These changes are inferred to have occurred exclusively on the ancestral branch of each of our four main clades, and therefore arose during the emergence of that clade. Of these, we detected significant evidence for positive selection (*d*_N_/*d*_S_ > 1; *p* < 0.01) on the Esculentum (red-fruited) group ancestral branch in 3.08% of genes (137 out of 4,447 testable genes; False Discovery Rate (FDR) = 32.5%), 4.69% in the Arcanum group (179 out of 3,819 genes; FDR = 21.3%), and 3.96% in the Hirsutum group (38 out of 958 genes; FDR 25.2%; see S1 Data 1.9–1.12 for all genes and *p*-values). Due to the variability in the gene tree topologies particular to the Peruvianum group, the ancestral branch appeared in only 10% of genes, so this group was not tested. Results for all genes tested, regardless of the presence of a lineage-specific nonsynonymous substitution, are presented in S1 Text Section 5.

In some instances, there are clear functional consequences for these group-specific amino acid changes. For example, all members of the red-fruited Esculentum group share such changes in 10 enzymes within the carotenoid biosynthesis pathway, which is responsible for red coloration (Fig 4C) [40,66–68]. Although not all elements of this pathway have been functionally characterized, current estimates are that it contains ~31 enzymes [67]; therefore, we find nearly a third of the enzymes in the carotenoid biosynthesis pathway have novel amino acid changes specific to the group that has evolved red-colored fruits. This includes four amino acid substitutions each in *Solyc06g036260* (*β-carotene hydroxylase 1*; *p* = 0.005) and *Solyc04g040190* (*lycopene β-cyclase 1*; *p* = 0.043). Other examples of adaptively evolving genes include 10 Arcanum-group-specific amino acid substitutions in *Solyc02g067670*, an ortholog of the *Arabidopsis* gene *UVR1* (*Ultraviolet Repair Defective 1*), which may be connected to adaptation to increased solar radiation at the high altitudes characteristic of these species (Fig 1; *p* < 10^−5^). We also found many putative species-specific substitutions across the tree, although more extensive intraspecific sampling will be required to confirm species-specificity. For example, both *S*. *chmielewskii* accessions shared six nonsynonymous changes in *Solyc06g051460* (ATP-dependent chaperone *ClpB*), a gene implicated in temperature stress response [69].

In addition to genes with obvious phenotypic consequences, these analyses also revealed group-specific loci with many amino-acid changes, but whose ecological functions are less clear. For example, five Esculentum-group-specific amino acid substitutions were observed in *Solyc09g082460* (a homocysteine S-methyltransferase, *p* = 4.64 × 10^−4^). This and other cases demonstrate the potential of this analysis to discover new candidate genes whose adaptive functional consequences are currently unknown, but that are intriguing targets for follow-up work (S1 Data 1.8–1.20).

Overall, across all of the loci for which we could test clade-specific sites, we found 3.8% of genes had evidence for positive selection (within PAML at *p* < 0.01) on at least one of our three well-supported branches. Though this number includes variable fractions of false positives (depending upon the branch involved), and we have conditioned on seeing lineage-specific nonsynonymous changes, it provides a crude estimate of the potential contribution of de novo mutation to new genetic variation in this clade.

### Environmental Selection Has Drawn from Ancestral Variation

In addition to lineage-specific changes, the close genetic relationships among wild tomato species make it possible to conduct a clade-wide, genome-wide investigation of genetic variants associated with broad-scale ecological factors (Fig 1) rather than shared genealogical history. Our expectation for this “PhyloGWAS” approach is that ancestrally segregating variants that confer an advantage to specific ecological conditions will be differentially fixed among current populations that share common environments, regardless of their phylogenetic relatedness. These genes will therefore show polyphyletic topologies that group species or accessions according to common environments. Note that this approach does not aim to detect molecular convergence (e.g., [70]), instead aiming to identify parallel selection on standing variation (e.g., [71]). Such surveys have been previously conducted within wild *S*. *lycopersicum* populations [72,73], but not among the clade as a whole. While the accessions used in our study were sampled from a broad geographic and environmental range (Fig 1), these analyses are only informative when ecological conditions are not confounded with phylogenetic relationships (i.e., when all members of a clade are not found in similar environments). This requirement excludes several broad ecological variables from testing, including variation in salinity, island versus mainland, and East versus West of the Andes. In addition, many potential environmental variables are highly correlated with each other, and data are often only available at relatively coarse environmental scales (S1 Text Section 6).

With these limitations in mind, we examined allelic associations with four ecological factors that were distributed among species within each of the major groups: altitude/temperature, a composite measure of seasonal climate variability, water pH, and soil heavy metal content. These factors capture broad axes of environmental variation among our samples while minimizing strongly correlated environmental variables (S1 Text Section 6). For all factors except altitude/temperature, we found numerous genes with environmentally associated alleles, and more loci than are expected to be environmentally associated by chance (see Materials and Methods, S1 Text Section 6), thereby generating a list of genes for which selection has putatively sorted functional allelic variants from variation ancestral to the entire group (S1 Data 1.22–1.25). Overall, we found 12 nonsynonymous variants (in 12 loci) associated with our second environmental factor (seasonal climate variation), 44 nonsynonymous variants (in 43 loci) associated with our third factor (soil pH), and 455 nonsynonymous variants (in 401 loci) associated with our fourth factor (variation in heavy metals). None of the loci identified to have nonsynonymous variation uniquely associated with differences in environmental factors is colocalized with a chromosomal region inferred to be introgressed between specific lineages. This indicates that loci putatively subject to selection from standing variation are not associated with inferred cases of cross-species introgression.

We found 12 genes with distinguishing amino acid differences between two groups of accessions that are found in distinct categories of seasonal climate variation (Fig 4C) described by a composite measure of latitudinal differences in temperature, precipitation seasonality, and the intensity of photosynthetically active radiation (PAR) (*p* < 2.5 × 10^−4^; S1 Text Section 6 and S1 Data 1.23). This list of genes includes several with potential roles in seasonal and latitudinal adaptation, including *Solyc02g069460* (photosystem I reaction center subunit III) and *Solyc12g014040* (chloroplast protein HCF243).

Even more strikingly, using mineral survey data from Peru to identify four populations sampled from habitats with high environmental levels of heavy metals (As, Cu, Hg, Ni, and Pb) and four from areas with low levels (Fig 4C), we found 401 genes with protein differences between the high and low metals groups (*p* < 2.5 × 10^−4^). These include a likely heavy metal binding/detoxification protein (*Solyc04g015030*), and two genes that require copper as a cofactor: *Solyc01g005510* (*Laccase-2*) and *Solyc08g079430* (*Primary amine oxidase*); these and other detected loci suggest that geographical variation in heavy metals in the Andean region may be a factor in local selection for functionally important ancestral variants. We also found environmentally sorted ancestral allelic variation associated with soil pH (S1 Text Section 6), enriched above that expected due to random association.

Note that, unlike in our cases of lineage-specific de novo adaptive evolution, these genes are generally characterized by only one or few nucleotide differences (S1 Data 1.22–1.25), as might be expected of alleles that are recruited from standing ancestral variation; that is, there is little reason to expect that functionally differentiated alleles would be segregating many sequence variants in the ancestral population. This small number of differences also makes it easier to determine whether introgression has contributed to the observed patterns of allele sharing. Under a model of introgression, we expect evidence for a localized block of variants that exhibit discordant phylogenetic signal regardless of whether changes are synonymous or nonsynonymous, whereas this is not expected for selection from standing variation. In our analysis, very few of the loci with environmentally associated nonsynonymous variants also had associated synonymous variants. For environmental factor 2 (seasonality), none of our candidate genes had synonymous variants in addition to the identified nonsynonymous variant (S1 Data 1.23). For factor 3 (soil pH), only 4 of 43 genes also had a single synonymous variant associated with the detected nonsynonymous variant(s). Associations between SNPs within candidate loci were slightly more common for environmental factor 4 (soil heavy metal content): of 401 genes with at least one environmentally associated nonsynonymous SNP, 55 loci also had 1 associated synonymous SNP. Of these 55 loci, 19 had >1 associated synonymous SNP. In these latter cases (~5%–14% of the identified candidates for this factor), we cannot unambiguously differentiate the relative contributions of standing variation and introgression. However, given the conditions of our tests of environmental association, it is unlikely that our detected candidates are frequently affected by introgression. This is because our tests for selection on standing variation explicitly required that variation in focal environmental factors be distributed among species and clades. For introgression to explain the distribution of these loci across distantly related (and often geographically distant) accessions would therefore require a mechanism involving multiple interspecific introgression events across different branches of the phylogeny, and in multiple geographical locations. Similarly, it is also unlikely that these results are generally explained by convergent de novo molecular changes, because each change would have to arise many times in many independent taxa, although we cannot exclude the possibility that some fraction of our loci might have been subject to convergence in one or a few taxa.

While the extensive shared variation detected in this group makes phylogenomic reconstruction much more complex, it also provides a novel opportunity to use a genome-wide association approach to identify candidate loci. Accordingly, in addition to lineage specific changes, we can point to potential examples of ecological selection on ancestral alleles as another mode of adaptation in this clade. Overall, across all the loci that could be compared for associations with our four environmental factors, 2.6% were found to have at least one nonsynonymous variant in perfect association with at least one of these factors (S1 Data 1.21–1.25), providing a provisional estimate of the potential for selection from standing variation across the clade.

### Multiple Genetic Sources and Ecological Factors Contribute to a Species Radiation

Lineages of closely related species can occupy diverse ecological roles, but the conditions that promote this rapid adaptive radiation are still under debate. Given multiple examples where only one of two closely related lineages experienced a burst of diversification under the same conditions, new ecological opportunity alone is likely to be insufficient [7,74–76]. This suggests that intrinsic factors—such as the availability of appropriate genetic variation—are equally critical for facilitating adaptive responses, although conditions that promote the origin and sharing of this variation remain largely speculative [77,78].

Here, we have found evidence for at least three significant sources of genetic variation that might facilitate adaptive diversification in response to ecological opportunity. First, we inferred introgression both between early lineages in the radiation and recently between specific populations. Second, we observed rapid lineage-specific adaptation from de novo mutation in genes related to functional traits that differ between groups. Finally, we find evidence of environment-specific sorting of ancestral variation. Analyses of other rapid radiations have also inferred the role of one or more of these three mechanisms in facilitating rapid diversification. For example, analyses of radiating African cichlids suggest widespread recruitment of potentially adaptive coding and regulatory variants from standing ancestral variation [7]. Clade-wide variation in Equids revealed evidence for both the rapid accumulation of de novo substitutions and for both ancient and recent introgression events between species [14]. In Darwin’s Finches [18], hybridization appears to play a role both in the origin of new lineages and potentially in the adaptive introgression of functional loci (e.g., for beak shape) between species. Because each of our analyses relies on different assumptions and varies in power, directly comparing the relative contribution of our three detected sources of genetic variation requires caution. Nonetheless, based on our crude estimates within each analysis, we infer that relatively small yet substantial fractions of the euchromatic genome are implicated in each source of genetic variation. We find little evidence that one of these processes predominates in its contribution, although our estimates suggest that de novo mutation might be relatively more influential and cross-species introgression relatively less so. This latter observation is in interesting contrast with several recent studies of animal adaptive radiations, including in Darwin’s Finches [18], Equids [14], and fish [13], where evidence suggests that hybridization and introgression might be much more pervasive and influential than previously suspected, and more abundant than we detect in *Solanum*. This is despite a greater historical emphasis on the role and importance of post-speciation gene flow in plant groups [79,80] and suggests that the dynamics of adaptive radiation might be less shaped by classical expectations of differences between broad taxonomic groups like plants and animals than expected. Rather, as with other studies that also detect one or more of these sources of genetic variation [7,14,17,18], we detect evidence for all three within the same diversifying clade, suggesting that these mechanisms may be universal in their facilitation of rapid adaptation to diverse environmental niches.

Rapid diversification via these three modes within wild tomatoes was likely ecologically driven by the extremely variable environments of the Andes and Galápagos. Notably, most of the significant geo-climatological transitions of this region substantially predate the entire history of wild tomato diversification. These events include major uplifts of the Central Andes [81–83] and the formation of biogeographic zones such as the Atacama Desert (at least ~14 Ma, though possibly up to ~150 Ma) and the Peruvian coastal desert [84,85]. Therefore, geographical and ecological expansion of wild tomato species was almost certainly due to migration into new environments rather than in situ adaptation during more ancient geological and climatic transitions. The timing of major lineage splits, in addition to the current distributions of extant species, can be used to infer the progression of these migratory steps (S1 Text Section 7.6 and S6 Fig). This south-to-north range expansion and diversification has been suggested by phylogenies of other plant and animal groups in the Central Andes [85–89]. More broadly, *Solanum* is one of the most speciose and widespread angiosperm genera, with ~1,500 extant species found on all continents except Antarctica. The last common ancestor of the genus is estimated to be only ~15.5 Ma [22,90–93]. Therefore, the rapid speciation rates that we see in the tomato clade, and the accompanying genetic and genomic changes, could be symptomatic of the factors facilitating sustained divergence and diversification across the entire *Solanum* genus around the globe.

## Materials and Methods

Full details of samples and methods are provided in S1 Text.

### Plant Material and Cultivation

Our sampling included 29 accessions from 13 species of tomato and two outgroup species (representing the entire clade and accepted outgroups; S1 Table). Seeds of each accession were obtained from the C. M. Rick Tomato Genetics Resource Center at the University of California, Davis (http://tgrc.ucdavis.edu). Seeds were germinated following standard guidelines (http://tgrc.ucdavis.edu) and then transplanted to 7.56-L pots containing a 1:1 mix of standard soil and Metro Mix 360 (http://www.hummert.com/) in the Department of Biology greenhouse at Indiana University under supplemental lighting to maintain a constant 14:10 h light:dark cycle. Plants were watered to field capacity daily to prevent drought stress and fertilized weekly.

### RNA Extraction and Library Preparation

To capture a wide set of transcripts, we harvested RNA from five different tissues: roots, leaf primordia and young/unexpanded leaves, mature leaves (fully expanded, the fifth leaf from the meristem), floral buds, and mature (open) unfertilized flowers. Tissue was collected in sterile 15 or 50 mL conical vials (VWR: 89039–666, 89039–658, respectively). Floral and leaf tissue was immediately placed into liquid nitrogen. Root tissue was washed in cold water for <60 s to remove large soil particles, blotted with paper towel for 10 s, and then frozen with liquid nitrogen. All tissues were pulverized under liquid nitrogen using a mortar and pestle; 50–100 mg fresh weight of ground tissue was used for total RNA extraction.

Extraction of the poly-A fraction of total RNA from ground tissue was performed using RNeasy Plant Mini Kits from Qiagen (catalog number 74904). Resuspended RNA was stored at −80°C until all samples were collected. Tissue-specific total RNA was equimolar pooled using the RiboGreen RNA quantitation assay (Life Technologies: R11491) and then quality checked using an Agilent 2200 TapeStation System prior to library construction. Stranded, paired-end libraries of total RNA were generated from these pools for each accession using Illumina TruSeq Stranded total RNA HT Sample Preparation Kits (Illumina: RS-122-2203), these libraries were pooled and distributed evenly (< 6-fold difference among libraries, S1 Data 1.1) across three lanes of Illumina HiSeqTM 2000 (Illumina Inc., San Diego, CA, US). RNA QC, library preparation, and pooling was performed by the Indiana University Center for Genomics and Bioinformatics (http://cgb.indiana.edu).

### RNA-Seq Read Processing and Mapping

Raw reads were filtered and trimmed using the SHEAR program (http://www.github.com/jbpease/shear). RNA-Seq reads were mapped to the *S*. *lycopersicum* reference genome v.SL2.50 (ftp://www.solgenomics.net) [40,41], reference chloroplast (NCBI accession NC_007898.3), and mitochondrial scaffolds (http://mitochondrialgenome.org/) using STAR [94]. Alignments were processed into multisample Variant Call Format (VCF) using SAMtools [95], then converted/filtered into Multisample Variant Format (MVF) using MVFtools (http://www.github.com/jbpease/mvftools) [45]. Two primary alignments were filtered: a high-quality (HQ) set requiring sequencing depth ≥ 3 and mapping quality ≥ 30, and a HD set with depth ≥ 10 and mapping quality ≥ 30 (see S1 Text Section 2.1–2.3 and S1 Data 1.1 for additional details).

### Phylogenetic Analysis

Phylogenies were inferred using several methods (RAxML [42], ASTRAL [21], MP-EST [20]) and partitions of the data. Using RAxML, whole-transcriptome and whole-chromosome concatenated phylogenies were inferred from all sites with alleles represented in ≥10 accessions. Molecular clock estimates were performed using *r8s* [96] with calibrated time points from Särkinen, Bohs [22]. RAxML was also used to infer phylogenies for 1 Mb and 100 kb genomic windows, and for annotated reference genes (ITAG v.2.4, https://www.solgenomics.net) with four or more accessions represented (S1 Text Sections 3.1–3.3).

From 100-kb window trees, a majority rule tree (S2D Fig) was computed using RAxML [42] and annotated with percentage of window tree support and IC/ICA scores for each node [44]. Coalescent trees were inferred with ASTRAL [21] and MP-EST [20] (S2E and S2F Fig). MP-EST was run for 100 replicates; the tree with the strongest likelihood score is shown in S2F Fig, as in [16]. Options for all three programs were set to default, and no consensus tree was used as input. RAxML was run with the “-J MRE” option for Majority Rule Extended. Majority rule and coalescent topologies agreed with the consensus phylogeny for the major subclades, with the exception of *hua*-1364 (see discussion below on Peruvianum group). The proportions of 100-kb trees supporting various nodes are shown in S2D Fig and S2 Table.

### Heterozygosity, Allele Variation, and Pairwise Distance Estimation

We calculated the proportion of heterozygous sites sampled from each accession and the patterns of alleles shared among groups from the HD alignment. Pairwise sequence distances between all pairs of the 29 sequenced accessions and the reference (S1 Data 1.2) were calculated from the HQ dataset using MVFtools. At heterozygous sites in an accession, one of the two alleles represented was selected randomly; random allele selection was also done for all analyses described below. Accessions in section *Lycopersicon* differ from accessions in *Lycopersicoides* by 2.10%–2.71% sequence divergence. Accessions within *Lycopersicon* have pairwise distances of 0.05%–1.7%, with the closest relationships between different accessions within *S*. *galapagense* (*gal*-3909/*gal*-0436) and within domesticated tomato (lyc-3475/*lyc*-ref) (S1 Text Section 3.2).

### Testing for Introgression

Using MVFtools [45], we calculated the *D*-statistic [51,59] for nonoverlapping 1-Mb windows of the HQcomp dataset, for all possible trios of the 27 *Lycopersicon* accessions and the reference. The consensus tree (Fig 2A) was used to determine expected tree topologies and to assign *P*_1_, *P*_2_, and *P*_3_. ABBA and BABA site patterns were combined for all windows to calculate a transcriptome-wide average *D*-statistic (S1 Data 1.5). Many cases were observed in which transcriptome-wide *D* values appeared to be driven almost entirely by a small number of 1 Mb windows, consistent with recent introgression at a localized chromosomal location against a background of generally low divergence (i.e., few ABBA and BABA patterns genome wide). To more directly assess whether the *D* values observed represented a genome-wide pattern of gene flow, we performed a bootstrap resampling analysis. For each trio of accessions, we randomly resampled 1 Mb windows with replacement and recomputed *D* (*n* = 10,000 replicates). From the distribution of resulting *D*-values, we assessed whether the 95% CI of the resampled distribution included *D* = 0.

From the *D*-statistics, we inferred putatively introgressing lineages (S4E and S4F Fig). We further investigated cases where trios of accessions showed evidence of widespread or significant amounts of introgression based on *D*-statistic calculations. For each putatively introgressed trio, we inferred gene trees for each protein-coding region using sequences from the trio and *lyd*-4126 as the outgroup (S1 Data 1.5, S4 and S5 Figs) using RAxML [42]. From these gene trees, we counted the proportion of gene trees of each of the three possible rooted topologies. In each introgression case, we estimated the proportion of genes that were introgressed as the difference in the proportions of trees with the two discordant topologies. In the case of the introgressions involving *neo-*2133, *neo*-1322, and *S*. *pimpinellifolium*, we also calculated *D*_FOIL_ statistics for 100 kb windows to infer the direction of introgression [52]; these cases involved tree topologies appropriate for the use of this 5-taxon method (S5A Fig). This window size is large enough to avoid problems associated with the sampling of trees from smaller windows [52,97].

### Test for Lineage-Specific Evolution

The high levels of incompletely sorted ancestral variation and variability of the gene-by-gene phylogenies presented a particular challenge to estimating genes with lineage- or species-specific substitutions. A standard tree-based *d*_N_/*d*_S_ model implicitly reconstructs ancestral states, which in our dataset would be subject to high error because of the pervasive background of ILS [65]. Instead, we used a more conservative variant of a *d*_N_/*d*_S_ test to infer which genes show high relative frequencies of nonsynonymous substitutions (and therefore are likely under positive selection) for the four well-supported subclades within *Lycopersicon* (Esculentum, Arcanum, Peruvianum, and Hirsutum groups), as well as some specific species (below). For a given gene, we counted only substitutions that could be placed unambiguously on the branch leading to a particular lineage. For example, when testing the Esculentum group as the target lineage of interest, substitutions were counted as lineage-specific only when the set of sites sampled from Esculentum group and the set of sites sampled from all other accessions (including the outgroup) were completely nonoverlapping in identity. These substitutions were tabulated as synonymous or nonsynonymous, depending on whether a change in amino acid occurred. For all tests, the outgroup accessions were included in the nontarget group. Sites were only considered when at least one allele was available for each ingroup species and at least one outgroup accession.

We tested for changes on the branch separating section *Lycopersicon* versus section *Lycopersicoides* and for all other samples against these groups/species: Esculentum group, Arcanum group, Peruvianum group, Hirsutum group, the Galápagos species, *S*. *pennellii*, *S*. *habrochaites*, *S*. *chilense*, *S*. *chmielewskii*, and *S*. *neorickii*. The domesticated accessions were not included since they have experienced intentional introgression of wild alleles for crop improvement. *hua-*1360 and *hua*-1364 were only included in the Peruvianum group-specific test because of their high incidence of reticulation.

We evaluated evidence for positive selection on the set of genes that showed lineage-specific substitutions for each of our well-supported branches (as outlined above) using the branch-site test in PAML 4.8a [98]. For each protein-coding gene, the codon alignment for that gene was extracted from the MVF-translated alignment file and accepted for testing only if at least one sequence was represented for each species (not including *hua*-1360 and *hua*-1364). To maximize the alignment tested, only the sequence for each ingroup species (among available accessions) with the most aligned codons represented was retained. Similarly, only the outgroup accession with the most aligned codons was also retained.

Phylogenies for each 14-species gene alignment were then inferred using RAxML v.8.1.16 [42] using standard parameters and the GTRGAMMA model. For each of the four major groups (Esculentum, Arcanum, Peruvianum, Hirsutum), we verified in the gene tree that all accessions in the given group are a monophyletic clade (i.e., that the gene tree has an appropriate branch ancestral to the group being tested). If this ancestral branch was not present, the gene was not tested for that particular group. Otherwise, the ancestral branch was marked as the target “foreground” branch and tested using the branch-site test in PAML. We ran both null and alternative tests, and recorded *d*_N_/*d*_S_ values and likelihood scores. Since branch lengths were fixed at the values provided by the RAxML tree, the null model has four free parameters and the alternative test has five. Therefore, significance was assessed by a likelihood ratio test (LRT) assuming a *χ* ^2^ distribution with one degree of freedom (see S1 Text Section 5 for full PAML control file parameters). From these tests, we calculated the proportion of genes that showed significance under the LRT (*p* < 0.01) both for (1) a set of genes where we had sampled at least one site where all accessions within the target group had alleles that differed from all accessions outside the target group (i.e., an exact allele pattern that indicated a nonsynonymous substitution on the branch leading to the target group; see Results) and (2) for all genes containing the target branch according to the RAxML gene trees (see S1 Text Section 5).

### Environmental Data

Geographical coordinates and sampling location information for each accession were obtained from the TGRC database (http://tgrc.ucdavis.edu). Altitude and temperature for each population location were extracted from the WorldClim database (www.worldclim.org); because many environmental factors in this database are strongly correlated across the natural range of wild tomatoes [26], we limited our analyses on WorldClim data to these two broadly representative factors. Soil solution pH data at 1 km resolution was obtained from the ISRIC SoilGrids project (http://soilgrids.org/).

Metal abundances for the Peruvian accessions were estimated from data in GEOCATMIN (http://geocatmin.ingemmet.gob.pe) provided by the Instituto Geologico Minero y Metalurgico de Peru. In combination with topographic and hydrological data from the same database, metal abundances (in ppm) were averaged for all sample points located within a 100 km^2^ centroid surrounding each accession’s coordinates, for sites directly upstream or downstream of the population location; this area corresponds to locations within ~11 km of each accession’s geographical location. Metal concentrations were taken from the “Geochemistry: Serie B: Prospecting Geochemistry Sediment ravine” survey data collected between 2002 and 2011.

PAR values for mainland South America were obtained from Insituto Nacional de Pesquisas Espaciais de Brasil (http://www.inpe.br/). These data are available in units of kWh/m^2^/d for 40-km resolution in monthly averages from data spanning 1995–2005. At this resolution, each accession inhabited a unique data cell except *hua-*1358 and *hua*-1360. PAR values were unavailable for Galápagos populations. Seasonality of PAR was estimated as the standard deviation of monthly averages.

### Test for Environment-Specific Sequence Differences

To identify genomic targets of selection in response to abiotic factors, we treated all the accessions in section *Lycopersicon* as a population and looked for alleles that differentiated environmentally classified populations in phylogenetic genome-wide association study (“PhyloGWAS”). These tests required that accessions from the same species or group occurred in different ecological categories, thus allowing detection of abiotic effects over lineage-specific effects. In our dataset, some environmental/geographical factors were intrinsically correlated with each other and thus were combined into a single composite environmental axis. Therefore, for our PhyloGWAS analysis, we selected four environmental axes that met the requirements for this approach: (1) altitude/temperature, (2) latitude/climate seasonality, (3) interpolated water pH, and (4) heavy metal abundance. In our sampled accessions, each environmental axis identified two clearly separable groups of populations (see S1 Text Section 6 for additional details).

For each of these four comparisons, we asked whether there were nonsynonymous variants completely correlated with each environmental condition. For instance, at a single position there might be an arginine present in all accessions experiencing high heavy metals, and a glycine present in all accessions in environments with low heavy metal concentrations. We examined all sites with nonsynonymous variants between any of the accessions used in each of the four environmental contrasts. This led to four sets of variants that were queried for environmental-specific changes; the size of the datasets examined were 233,567 nonsynonymous variants for abiotic Factor 1, 253,161 for Factor 2, 160,255 for Factor 3, and 198,908 for Factor 4. We found nonsynonymous variants perfectly associated with our environmental factors for all four contrasts, except for Factor 1 (altitude/temperature), which was nonetheless associated with three synonymous variants. The numbers observed were 0 nonsynonymous variants for Factor 1, 12 for Factor 2 (in 12 genes), 44 for Factor 3 (in 43 genes), and 455 for Factor 4 (in 401 genes).

To assess the significance of observing these patterns, we used the program ms [99] to simulate 10^9^ genes with a single variable site over the consensus phylogeny (Fig 2A) using *N*_*e*_ = 10^5^ and 2.5 generations per year. For each environmental factor, we determined the number of times we could expect a perfect association between variants and the environment due to ILS alone, out of the specific number of variants examined for that contrast. To do this, we simulated many datasets of the same size as the ones we tested, and for each dataset recorded the number of perfectly associated variants observed. The *p*-values for all four environmental contrasts are the proportion of simulated datasets that have a greater number of genes perfectly associated with environmental variables than our observed values (see S1 Text Section 6 for additional details).

### Software and Data Access

MVFtools is freely available at http://www.github.com/jbpease/mvftools. Plots were generated with the Python *matplotlib* (http://www.matplotlib.org) and Veusz (http://home.gna.org/veusz/). Phylogenies were prepared with FigTree (http://tree.bio.ed.ac.uk/software/figtree/). “Cloudogram” diagrams were generated with DensiTree (https://www.cs.auckland.ac.nz/~remco/DensiTree/). All other analyses were performed with custom Python scripts, using the BioPython, NumPy, and SciPy libraries. Read trimming, mapping, and large-scale file conversions were performed on the Mason High Performance Computing Cluster at Indiana University.

All raw sequence reads are available on NCBI SRA at Bioproject PRJNA305880. VCF, MVF, and phylogeny files are deposited in the Dryad repository http://dx.doi.org/10.5061/dryad.182dv [100]. The tomato reference genome is available from SolGenomics (http://www.solgenomics.net). List of Ultra-Conserved Orthologs can be found at the *Compositae* Genome Project (http://compgenomics.ucdavis.edu). Additional geographic, ecological, and sampling information on the accessions used in this study is available at http://www.tgrc.ucdavis.edu.

## Supporting Information

S1 Data Open Document spreadsheet file containing raw data statistics, sequence statistics, lineage-specific and environmental gene lists, environmental information, and phylogenetic discordance data. (XLSX)

S1 Fig Species ranges of *Solanum* sections *Lycopersicon*, *Lycopersicoides*, and *Juglandifolia*. Estimated species ranges for all species inferred from wild accession collection sites in the Tomato Genetics Resource Center (http://tgrc.ucdavis.edu). Red and pink arrowheads on the Galápagos Islands indicate island accessions of *S*. *lycopersicum* and *S*. *pimpinellifolium*, respectively. Species from the closely related outgroup section *Juglandifolia* (*S*. *juglandifolium* and *S*. *ochranthum*) were not sequenced for this study, but are shown here for completeness (base map modified from original from http://www.freevectormaps.com
(PDF)

S2 Fig Whole-transcriptome phylogenies. (A) Whole-transcriptome concatenated phylogeny (RAxML) including the reticulate lineage *hua*-1360, which was excluded from the species phylogeny in Fig 2. (B) Whole-transcriptome concatenated phylogeny (RAxML) with a single outgroup accession and excluding domesticated (*lyc*-ref and *lyc*-3475), putative reticulate (*hua*-1360, *hua*-1364, *per*-2744), and low coverage (*chm*-1028) accessions. (C) Whole-transcriptome concatenated phylogeny (RAxML) inferred using only Peruvianum group accessions and the outgroup. (D) Majority rule phylogeny (RAxML) using 100 kb genomic window phylogenies with percentage of trees supporting each node, and IC/ICA scores. (*E*) Coalescent-based phylogeny (ASTRAL) using 100-kb genomic windows. (*F*) Best likelihood coalescent-based phylogeny from 100 replicates of MP-EST using 100 kb genome window trees.
(PDF)

S3 Fig Ancestral allele sorting. (A) The variation patterns of 12.1 million HD aligned sites are shown, including variation within the tomato clade and among accessions, species, and major groups. (B) The number of heterozygous sites and rate of heterozygous sites per accession sampled for each group. Grey boxes (middle sections) show the number of sites sorting the same two ancestral alleles among 2 or 3 groups. 693 sites are sorting in all four groups. (C) Comparison of branch lengths versus branch concordance from the consensus concatenated phylogeny (Fig 2A) showing a positive correlation between branch length and the proportion of 100-kb window trees that include a given branch. Colors match group colors from Fig 2 with black for backbone phylogeny branches and grey for the outgroup.
(PDF)

S4 Fig Reticulate lineages and ancestral introgressions. (A) The four most frequent gene trees are shown for quartets using *chi*-4117A, *hua*-1360, *pim*-1589, and *neo*-1322, with *lyd*-4126 as outgroup. (B) Chromoplot showing the spatial distribution of 100 kb genomic window phylogenies for *hua*-1360, *per*-2964, and *pim*-1589, with gene trees and proportions shown. The proportions of sites where *hua*-1360 is fixed for Peruvianum- or Esculentum/Arcanum-specific alleles are shown (right). (C–D) Chromoplots, gene trees, and allele proportions similar to (B) for *hua*-1364 and *per*-2744. (E–F) Chromoplots and gene tree proportions for inferred ancestral introgressions indicated on the simplified species trees (right side).
(PDF)

S5 Fig Recent introgressions. (A) *D*_FOIL_ statistics for 100 kb windows on the short arm of chromosome 1 for the taxa and tree shown (left side). Shaded regions indicate *D*_FOIL_ signatures for the directional introgressions shown with *p* < 0.001 for all *D*_FOIL_ components. (B–D) Chromoplots showing the spatial distribution of phylogenies inferred from 100 kb regions, gene tree proportions, and enlarged regions (highlighted by boxes on the chromoplots) show sites of putative recent introgression. Annotation show approximate boundaries using the SL2.50 reference coordinates and the approximate bounding genes that encompass the introgressed region. (E) Chromoplots and gene tree proportions for three recently introgressing accessions from the Peruvianum group. Dark bars added to visually highlight regions with a strong local enrichment of a discordant phylogeny that indicates introgression.
(PDF)

S6 Fig Hypothesis for the geographic spread and diversification of *Lycopersicon* lineages. Arrows indicate the most likely routes for species or groups, with annotations at major hypothesized transitions. For full description, see S1 Text Section 7.6 (base map modified from original from http://www.freevectormaps.com)
(PDF)

S1 Table *Solanum* accessions used in this study. (DOCX)

S2 Table Support for clades from 100kb and 1-Mb windows, reference genes, and chromosomes. (DOCX)

S1 Text Supporting Information containing additional details of methods and results. (DOCX)

S2 Text Abstract en Español (Abstract in Spanish). (DOCX)

## Abbreviations

HD: high-depth
HQ: high-quality
ILS: incomplete lineage sorting
LRT: likelihood ratio test
Ma: million years ago
MVF: Multisample Variant Format
PAR: photosynthetically active radiation
VCF: Variant Call Format
