# Supplementary figures and images for: Phylogenomics Reveals Three Sources of Adaptive Variation during a Rapid Radiation
Source: PLoS Biol. 2016 Feb 12;14(2):e1002379. doi: 10.1371/journal.pbio.1002379 (PMC4752443; doi:10.1371/journal.pbio.1002379)

Figure S1

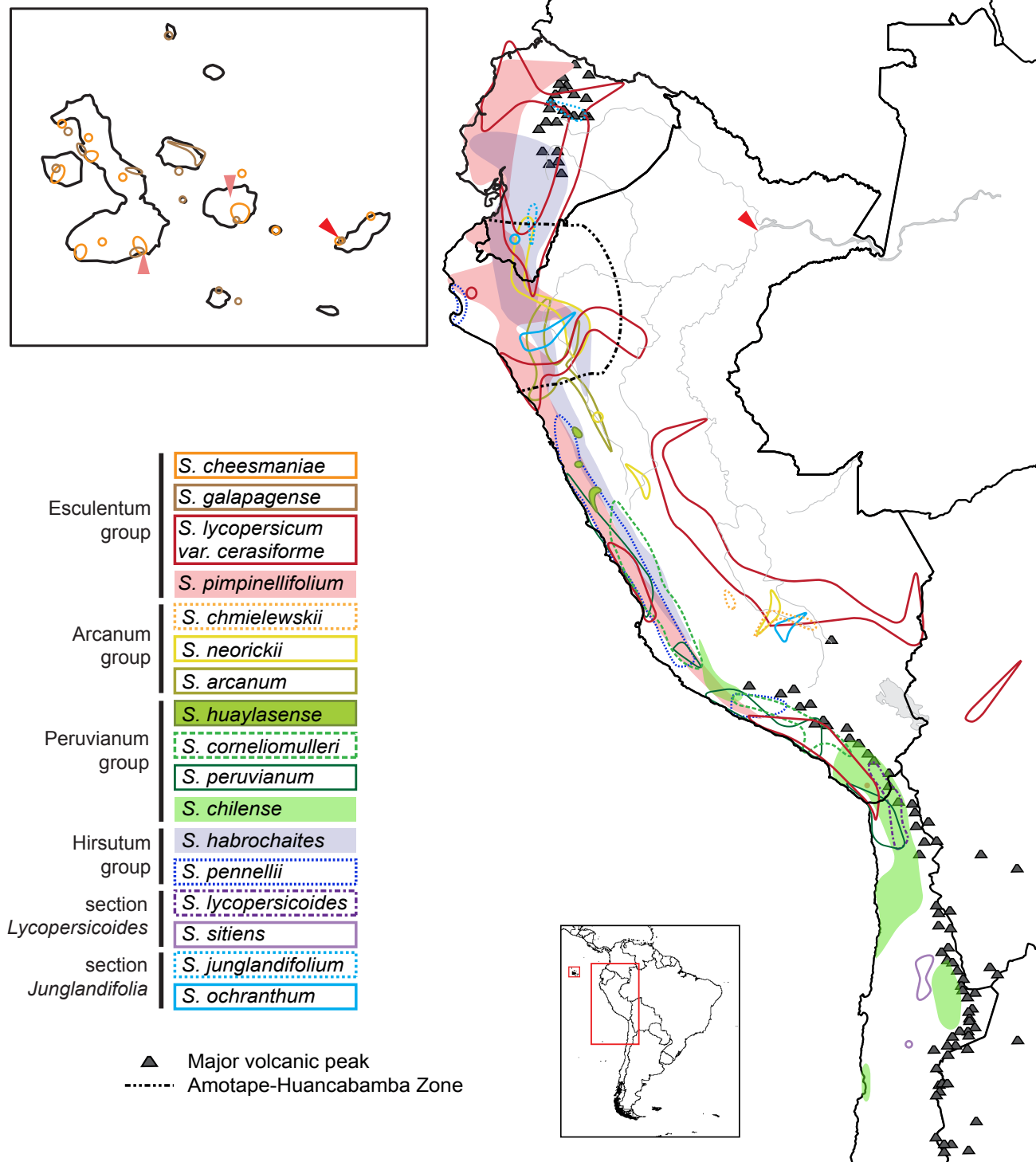

Supplement: S1 Fig — Estimated species ranges for all species inferred from wild accession collection sites in the Tomato Genetics Resource Center (http://tgrc.ucdavis.edu). Red and pink arrowheads on the Galápagos Islands indicate island accessions of S. lycopersicum and S. pimpinellifolium, respectively. Species from the closely related outgroup section Juglandifolia (S. juglandifolium and S. ochranthum) were not sequenced for this study, but are shown here for completeness (base map modified from original from http://www.freevectormaps.com (PDF) [file pbio.1002379.s002.pdf]

Figure S2

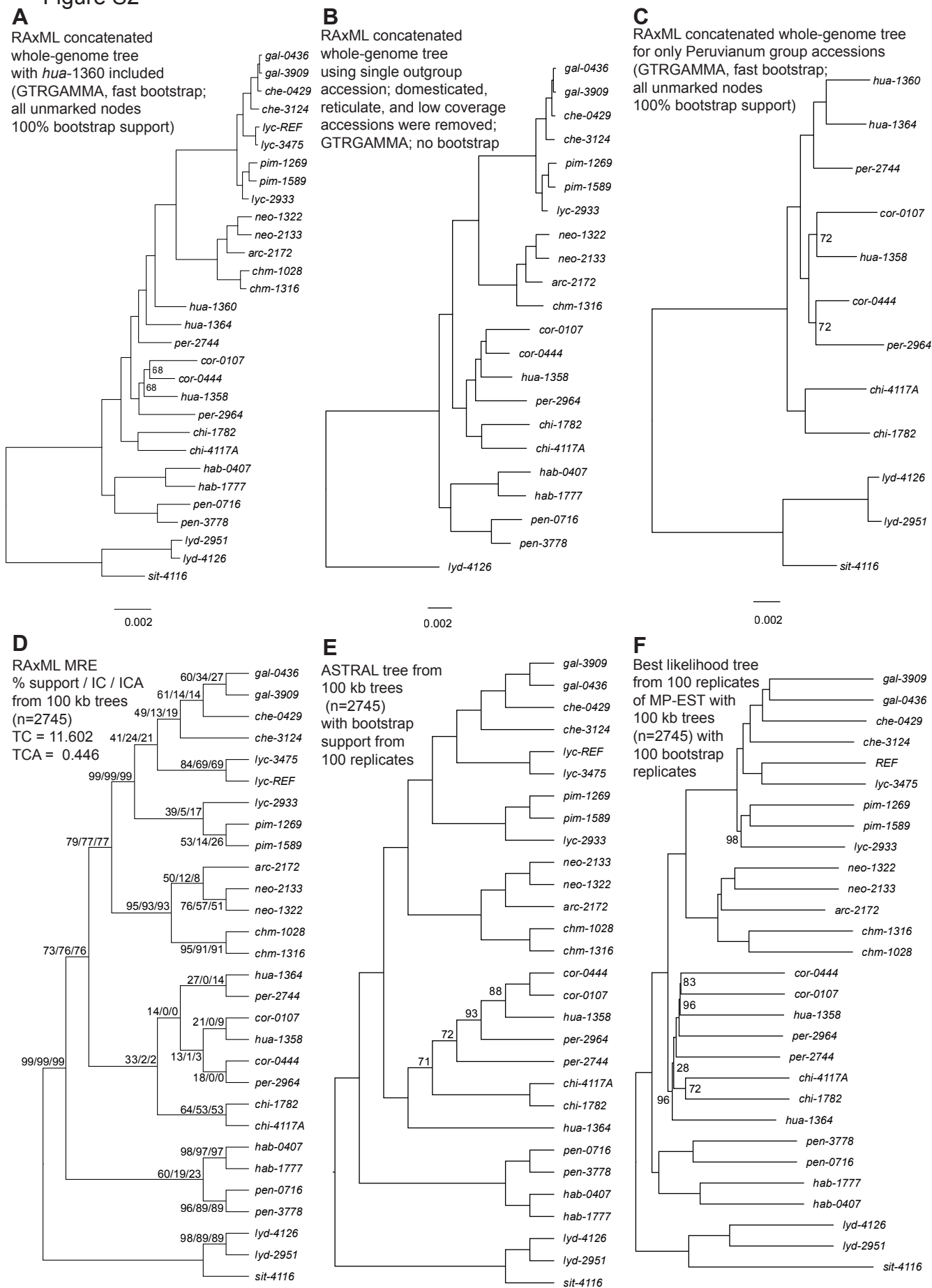

Supplement: S2 Fig — (A) Whole-transcriptome concatenated phylogeny (RAxML) including the reticulate lineage hua-1360, which was excluded from the species phylogeny in Fig 2. (B) Whole-transcriptome concatenated phylogeny (RAxML) with a single outgroup accession and excluding domesticated (lyc-ref and lyc-3475), putative reticulate (hua-1360, hua-1364, per-2744), and low coverage (chm-1028) accessions. (C) Whole-transcriptome concatenated phylogeny (RAxML) inferred using only Peruvianum group accessions and the outgroup. (D) Majority rule phylogeny (RAxML) using 100 kb genomic window phylogenies with percentage of trees supporting each node, and IC/ICA scores. (E) Coalescent-based phylogeny (ASTRAL) using 100-kb genomic windows. (F) Best likelihood coalescent-based phylogeny from 100 replicates of MP-EST using 100 kb genome window trees. (PDF) [file pbio.1002379.s003.pdf]

**Figure S3****A**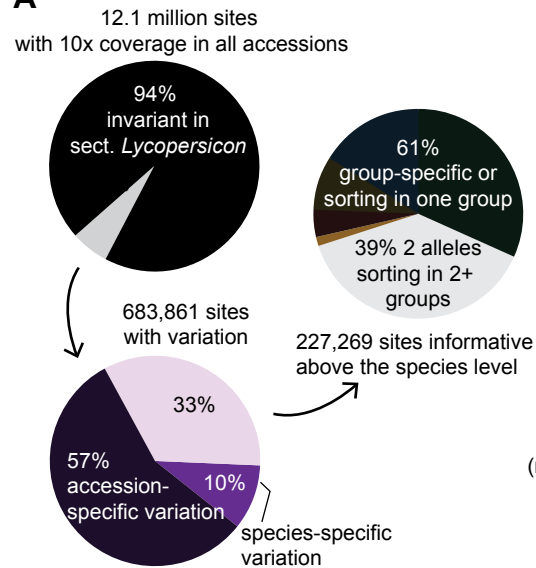**B**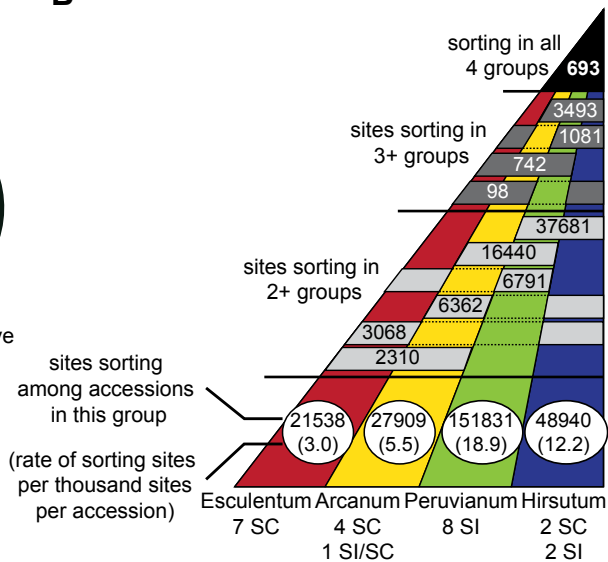**C**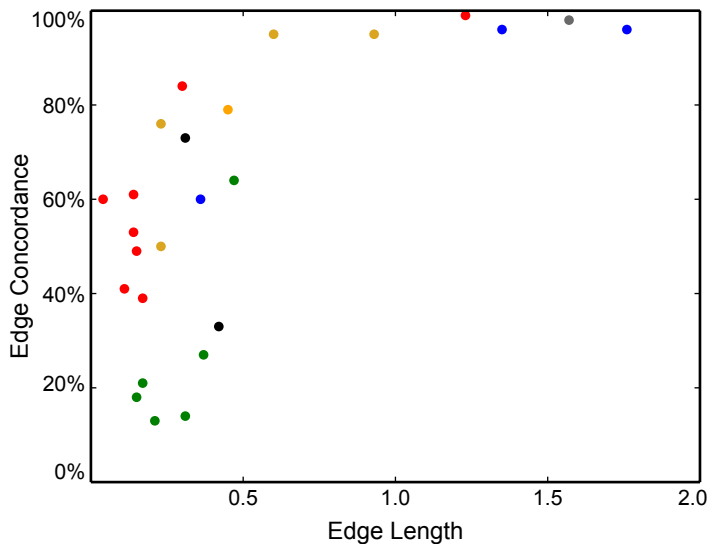

Supplement: S3 Fig — (A) The variation patterns of 12.1 million HD aligned sites are shown, including variation within the tomato clade and among accessions, species, and major groups. (B) The number of heterozygous sites and rate of heterozygous sites per accession sampled for each group. Grey boxes (middle sections) show the number of sites sorting the same two ancestral alleles among 2 or 3 groups. 693 sites are sorting in all four groups. (C) Comparison of branch lengths versus branch concordance from the consensus concatenated phylogeny (Fig 2A) showing a positive correlation between branch length and the proportion of 100-kb window trees that include a given branch. Colors match group colors from Fig 2 with black for backbone phylogeny branches and grey for the outgroup. (PDF) [file pbio.1002379.s004.pdf]

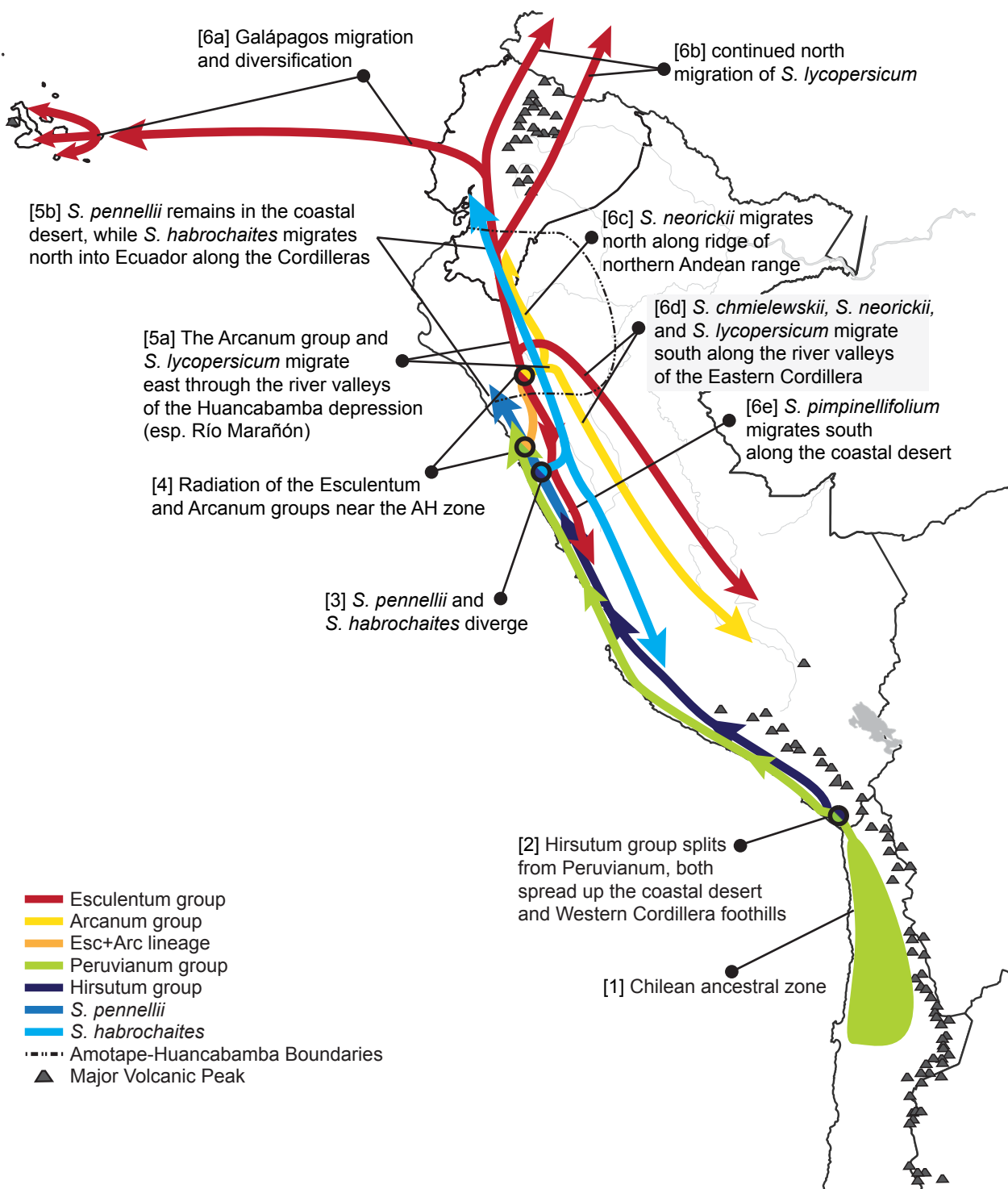

Supplement: S6 Fig — Arrows indicate the most likely routes for species or groups, with annotations at major hypothesized transitions. For full description, see S1 Text Section 7.6 (base map modified from original from http://www.freevectormaps.com) (PDF) [file pbio.1002379.s007.pdf]
