# Supplementary material for: Phylogenomics Reveals Three Sources of Adaptive Variation during a Rapid Radiation
Source: PLoS Biol. 2016 Feb 12;14(2):e1002379. doi: 10.1371/journal.pbio.1002379 (PMC4752443; doi:10.1371/journal.pbio.1002379)
Supplement: S4 Fig — (A) The four most frequent gene trees are shown for quartets using chi-4117A, hua-1360, pim-1589, and neo-1322, with lyd-4126 as outgroup. (B) Chromoplot showing the spatial distribution of 100 kb genomic window phylogenies for hua-1360, per-2964, and pim-1589, with gene trees and proportions shown. The proportions of sites where hua-1360 is fixed for Peruvianum- or Esculentum/Arcanum-specific alleles are shown (right). (C–D) Chromoplots, gene trees, and allele proportions similar to (B) for hua-1364 and per-2744. (E–F) Chromoplots and gene tree proportions for inferred ancestral introgressions indicated on the simplified species trees (right side). (PDF) [file pbio.1002379.s005.pdf]

Figure S4

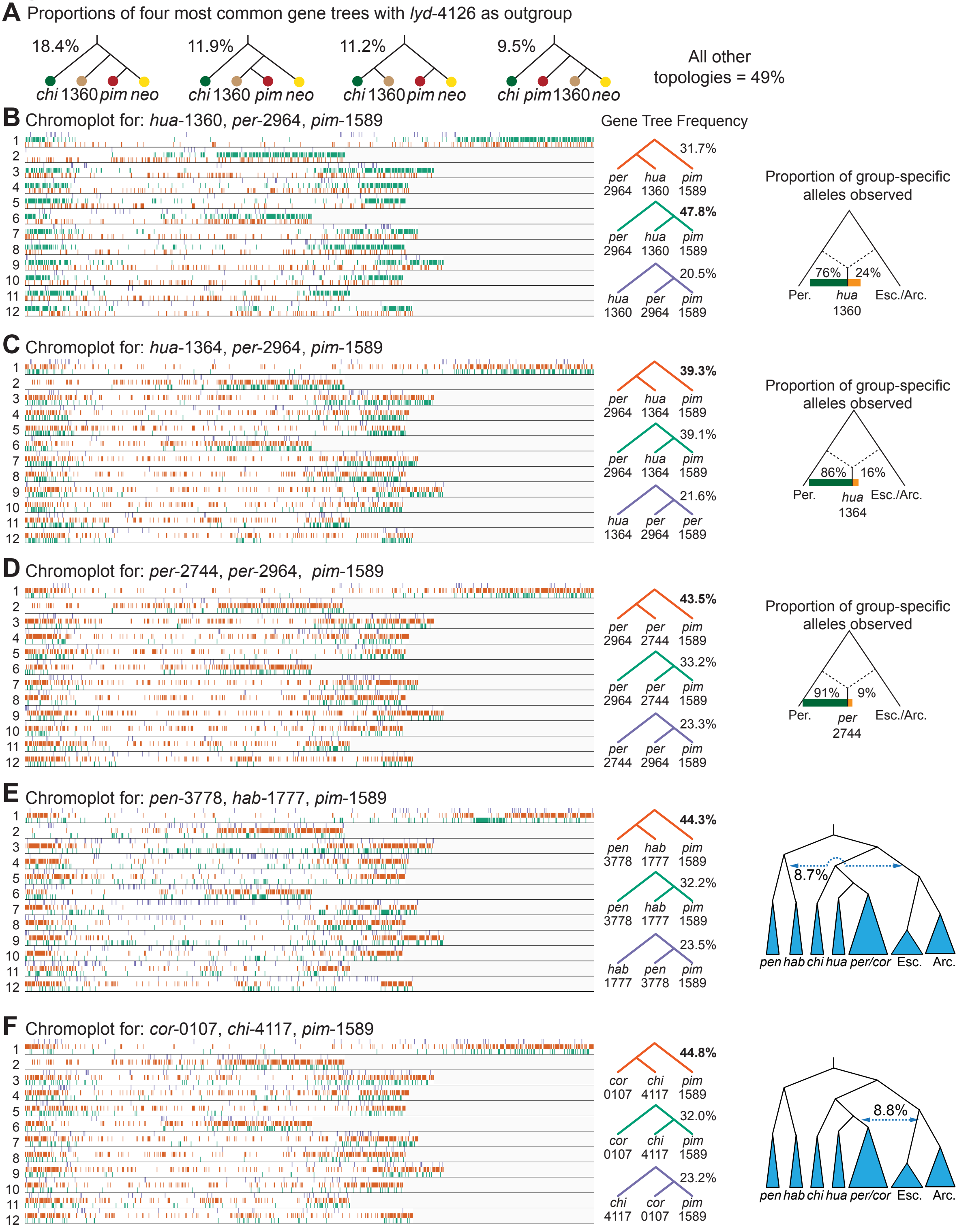

A Proportions of four most common gene trees with lyd-4126 as outgroup
18.4%
chi 1360 pim neo
11.9%
chi 1360 pim neo
11.2%
chi 1360 pim neo
9.5%
chi pim 1360 neo
All other topologies = 49%
B Chromoplot for: hua-1360, per-2964, pim-1589
Gene Tree Frequency
31.7%
per 2964 hua 1360 pim 1589
47.8%
per 2964 hua 1360 pim 1589
20.5%
hua 1360 per 2964 pim 1589
Proportion of group-specific alleles observed
76% 24%
Per. hua 1360 Esc./Arc.
C Chromoplot for: hua-1364, per-2964, pim-1589
39.3%
per 2964 hua 1364 pim 1589
39.1%
per 2964 hua 1364 pim 1589
21.6%
hua 1364 per 2964 per 1589
Proportion of group-specific alleles observed
86% 16%
Per. hua 1364 Esc./Arc.
D Chromoplot for: per-2744, per-2964, pim-1589
43.5%
per 2964 per 2744 pim 1589
33.2%
per 2964 per 2744 pim 1589
23.3%
per 2744 per 2964 pim 1589
Proportion of group-specific alleles observed
91% 9%
Per. per 2744 Esc./Arc.
E Chromoplot for: pen-3778, hab-1777, pim-1589
44.3%
pen 3778 hab 1777 pim 1589
32.2%
pen 3778 hab 1777 pim 1589
23.5%
hab 1777 pen 3778 pim 1589
8.7%
pen hab chi hua per/cor Esc. Arc.
F Chromoplot for: cor-0107, chi-4117, pim-1589
44.8%
cor 0107 chi 4117 pim 1589
32.0%
cor 0107 chi 4117 pim 1589
23.2%
chi 4117 cor 0107 pim 1589
8.8%
pen hab chi hua per/cor Esc. Arc.
