# Supplementary material for: Phylogenomics Reveals Three Sources of Adaptive Variation during a Rapid Radiation
Source: PLoS Biol. 2016 Feb 12;14(2):e1002379. doi: 10.1371/journal.pbio.1002379 (PMC4752443; doi:10.1371/journal.pbio.1002379)
Supplement: S5 Fig — (A) D FOIL statistics for 100 kb windows on the short arm of chromosome 1 for the taxa and tree shown (left side). Shaded regions indicate D FOIL signatures for the directional introgressions shown with p < 0.001 for all D FOIL components. (B–D) Chromoplots showing the spatial distribution of phylogenies inferred from 100 kb regions, gene tree proportions, and enlarged regions (highlighted by boxes on the chromoplots) show sites of putative recent introgression. Annotation show approximate boundaries using the SL2.50 reference coordinates and the approximate bounding genes that encompass the introgressed region. (E) Chromoplots and gene tree proportions for three recently introgressing accessions from the Peruvianum group. Dark bars added to visually highlight regions with a strong local enrichment of a discordant phylogeny that indicates introgression. (PDF) [file pbio.1002379.s006.pdf]

Figure S5

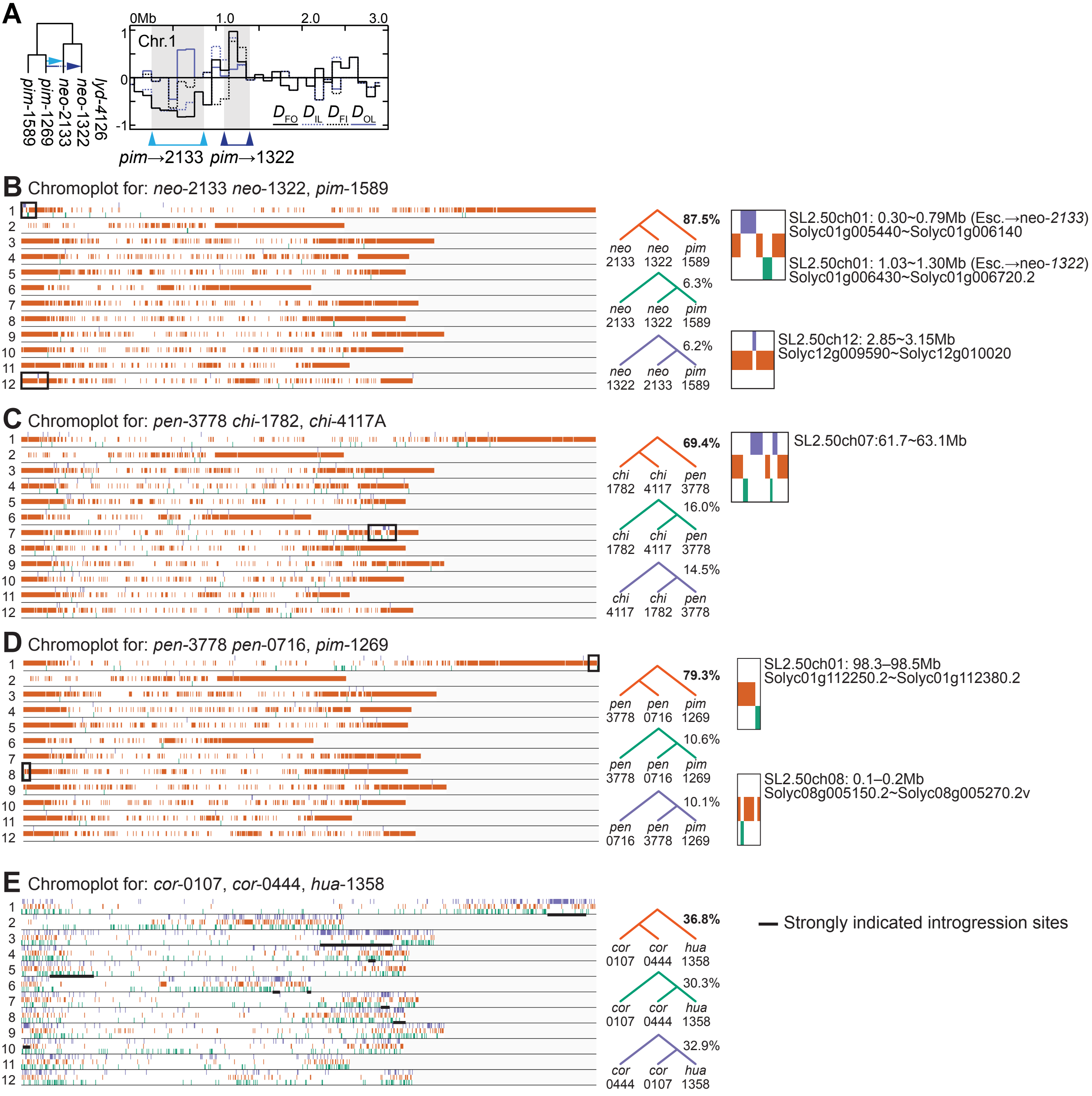

A
0Mb
1.0
2.0
3.0
Chr.1
1
0
-1
pim-1589
pim-1269
neo-2133
neo-1322
lyd-4126
D_{FO} D_{IL} D_{FI} D_{OL}
pim→2133 pim→1322
B Chromoplot for: neo-2133 neo-1322, pim-1589
87.5%
neo 2133 neo 1322 pim 1589
6.3%
neo 2133 neo 1322 pim 1589
6.2%
neo 1322 neo 2133 pim 1589
SL2.50ch01: 0.30~0.79Mb (Esc.→neo-2133)
Solyc01g005440~Solyc01g006140
SL2.50ch01: 1.03~1.30Mb (Esc.→neo-1322)
Solyc01g006430~Solyc01g006720.2
SL2.50ch12: 2.85~3.15Mb
Solyc12g009590~Solyc12g010020
C Chromoplot for: pen-3778 chi-1782, chi-4117A
69.4%
chi 1782 chi 4117 pen 3778
16.0%
chi 1782 chi 4117 pen 3778
14.5%
chi 4117 chi 1782 pen 3778
SL2.50ch07:61.7~63.1Mb
D Chromoplot for: pen-3778 pen-0716, pim-1269
79.3%
pen 3778 pen 0716 pim 1269
10.6%
pen 3778 pen 0716 pim 1269
10.1%
pen 0716 pen 3778 pim 1269
SL2.50ch01: 98.3–98.5Mb
Solyc01g112250.2~Solyc01g112380.2
SL2.50ch08: 0.1–0.2Mb
Solyc08g005150.2~Solyc08g005270.2v
E Chromoplot for: cor-0107, cor-0444, hua-1358
36.8%
cor 0107 cor 0444 hua 1358
30.3%
cor 0107 cor 0444 hua 1358
32.9%
cor 0444 cor 0107 hua 1358
Strongly indicated introgression sites
