# Supplementary material for: Phylogenomics Reveals Three Sources of Adaptive Variation during a Rapid Radiation
Source: PLoS Biol. 2016 Feb 12;14(2):e1002379. doi: 10.1371/journal.pbio.1002379 (PMC4752443; doi:10.1371/journal.pbio.1002379)
Supplement: S1 Table — (DOCX) [file pbio.1002379.s008.docx]

## S1 Table: *Solanum* accessions used in this study

| **Accession** | **Species** | ***Section*/Group** | **Latitude** | **Longitude** | **Region** |
| --- | --- | --- | --- | --- | --- |
| LA0107 | *S. corneliomulleri* | Peruvianum | −13.017 | −76.383 | Peru |
| LA0407 | *S. habrochaites* | Hirsutum | −2.1781 | −79.914 | Ecuador |
| LA0429 | *S. galapagense* | Esculentum | −0.6439 | −90.329 | Galápagos |
| LA0436 | *S. cheesmaniae* | Esculentum | −0.9525 | −90.978 | Galápagos |
| LA0444 | *S. corneliomulleri* | Peruvianum | −13.433 | −76.133 | Peru |
| LA0716 | *S. pennellii* | Hirsutum | −16.225 | −73.617 | Peru |
| LA1028 | *S. chmielewskii* | Arcanum | −13.883 | −73.017 | Peru |
| LA1269 | *S. pimpinellifolium* | Esculentum | −11.475 | −77.108 | Peru |
| LA1316 | *S. chmielewskii* | Arcanum | −13.393 | −73.915 | Peru |
| LA1322 | *S. neorickii* | Arcanum | −13.45 | −72.43 | Peru |
| LA1358 | *S. huaylasense* | Peruvianum | −9.5333 | −77.967 | Peru |
| LA1360 | *S. huaylasense* | Peruvianum | −9.5469 | −77.93 | Peru |
| LA1364 | *S. huaylasense* | Peruvianum | −10.133 | −77.392 | Peru |
| LA1589 | *S. pimpinellifolium* | Esculentum | −8.39 | −78.74 | Peru |
| LA1777 | *S. habrochaites* | Hirsutum | −9.55 | −77.667 | Peru |
| LA1782 | *S. chilense* | Peruvianum | −15.367 | −74.625 | Peru |
| LA2133 | *S. neorickii* | Arcanum | −3.4 | −79.183 | Ecuador |
| LA2172 | *S. arcanum* | Arcanum | −6.0006 | −78.908 | Peru |
| LA2744 | *S. peruvianum* | Peruvianum | −18.55 | −70.15 | Chile |
| LA2933 | *S. lycopersicum* | Esculentum | −1.3333 | −80.583 | Ecuador |
| LA2951 | *S. lycopersicoides* | *Lycopersicoides* | −19.317 | −69.45 | Chile |
| LA2964 | *S. peruvianum* | Peruvianum | −18.028 | −70.835 | Peru |
| LA3124 | *S. cheesmaniae* | Esculentum | −0.8039 | −90.042 | Galápagos |
| LA3475 | *S. lycopersicum* | Esculentum | (domesticated) | | |
| LA3778 | *S. pennellii* | Hirsutum | −14.775 | −75.034 | Peru |
| LA3909 | *S. galapagense* | Esculentum | −0.2806 | −90.552 | Galápagos |
| LA4116 | *S. sitiens* | *Lycopersicoides* | −22.159 | −68.782 | Chile |
| LA4117A | *S. chilense* | Peruvianum | −22.908 | −67.941 | Chile |
| LA4126 | *S. lycopersicoides* | *Lycopersicoides* | −19.287 | −69.396 | Chile |
