# Supplementary material for: Phylogenomics Reveals Three Sources of Adaptive Variation during a Rapid Radiation
Source: PLoS Biol. 2016 Feb 12;14(2):e1002379. doi: 10.1371/journal.pbio.1002379 (PMC4752443; doi:10.1371/journal.pbio.1002379)
Supplement: S2 Table — (DOCX) [file pbio.1002379.s009.docx]

## S2 Table: Support for clades from 100 kb and 1 Mb windows, reference genes and chromosomes

| **species/group (# accessions)** | **# trees where species/group accessions are monophyletic** | | | |
| --- | --- | --- | --- | --- |
|  | **100 kb trees** | **1 Mb trees** | **Reference gene trees (including *hua*-1360)** | **Chromosome trees** |
| **TOTAL** | **2745** | **343** | **12237** | **12** |
| **Esculentum group (7)** | **2723 (99.2%)** | **341 (99.4%)** | **11999 (98.0%)** | **12** |
| **Galápagos species (4)** | **1518 (55.3%)** | **199 (58.0%)** | **7458 (60.9%)** | **12** |
| *S. cheesmaniae* (2) | 665 (24.2%) | 62 (18.1%) | 1812 (14.8%) | 0 |
| *S. galapagense* (2) | 1656 (59.0%) | 303 (88.3%) | 4000 (32.7%) | 12 |
| *S. lycopersicum* (3) | 858 (31.3%) | 28 (8.2%) | 6912 (56.5%) | 0 |
| *S. pimpinellifolium* (2) | 1460 (53.2%) | 221 (64.4%) | 4499 (36.7%) | 9 (not 1,4,9) |
| **Arcanum group (5)** | **2608 (95.0%)** | **339 (98.8%)** | **9574 (78.2%)** | **12** |
| *S. chmielewskii* (2) | 2608 (95.0%) | 340 (99.1%) | 8673 (70.8%) | 12 |
| *S. neorickii* (2) | 2077 (75.7%) | 326 (95.0%) | 5693 (46.5%) | 12 |
| *S. neo. + S. arcanum* (3) | 1371 (49.9%) | 233 (67.9%) | 4711 (38.4%) | 12 |
| **Peruvianum group (9)** | **923 (33.6%)** | **197 (57.4%)** | **821 (6.7%)** | **12** |
| *S. chilense* (2) | 1747 (63.6%) | 305 (88.9%) | 3450 (28.2%) | 12 |
| *S. corneliomulleri* (2) | 576 (21.0%) | 112 (32.7%) | 1603 (13.1%) | 5 (2,4-6,9) |
| *S. huaylasense* (2/3*) | 155 (5.6%) | 9 (2.6%) | 586 (4.8%) | 0 |
| *S. peruvianum* (2) | 162 (6.1%) | 17 (5.0%) | 721 (5.9%) | 0 |
| **Hirsutum group (4)** | **1669 (60.8%)** | **260 (75.8%)** | **4222 (34.5%)** | **12** |
| *S. habrochaites* (2) | 2694 (98.1%) | 341 (99.4%) | 10151 (82.9%) | 12 |
| *S. pennellii* (2) | 2641 (96.2%) | 341 (99.4%) | 8923 (72.9%) | 12 |

**^*^***hua*-1360 was used in the construction of gene trees but not genomic window trees
