# Supplementary material for: Phylogenomics Reveals Three Sources of Adaptive Variation during a Rapid Radiation
Source: PLoS Biol. 2016 Feb 12;14(2):e1002379. doi: 10.1371/journal.pbio.1002379 (PMC4752443; doi:10.1371/journal.pbio.1002379)
Supplement: S1 Text — (DOCX) [file pbio.1002379.s010.docx]

Supporting Information: S1 Text

**Phylogenomics reveals three sources of adaptive variation during a rapid radiation**

James B. Pease, David C. Haak, Matthew W. Hahn, Leonie C. Moyle.

[S1 Text Section 1: Terminology 1](#_Toc438152000)

[1.1: Taxonomy 1](#_Toc438152001)

[1.2: Species Groups 2](#_Toc438152002)

[S1 Text Section 2: Sequencing 2](#_Toc438152003)

[2.1: Sequencing and Read Processing 2](#_Toc438152004)

[2.2: Genome Preparation for Mapping: 3](#_Toc438152005)

[2.3: RNA-Seq mapping and Alignment Processing: 3](#_Toc438152006)

[2.4: *de novo* assembly of unmapped reads 4](#_Toc438152007)

[S1 Text Section 3: Phylogenetic and genetic distance analyses 4](#_Toc438152008)

[3.1: Whole-transcriptome, chromosome, genomic window and gene phylogenies 4](#_Toc438152009)

[3.2: Heterozygosity and Pairwise *d*_N_ and *d*_S_ 5](#_Toc438152010)

[3.3: Divergence times 6](#_Toc438152011)

[3.4: Chloroplast and mitochondrial sequences 6](#_Toc438152012)

[S1 Text Section 4: Introgression analyses 6](#_Toc438152013)

[S1 Text Section 5: Lineage-specific evolution analyses 7](#_Toc438152014)

[S1 Text Section 6: PhyloGWAS analyses 8](#_Toc438152015)

[S1 Text Section 7: Phylogeny of *Solanum* section *Lycopersicon* and major groupings 10](#_Toc438152016)

[7.1: Sections Lycopersicon and Lycopersicoides 10](#_Toc438152017)

[7.2: Hirsutum group 10](#_Toc438152018)

[7.3: Esculentum group 11](#_Toc438152019)

[7.4: Arcanum group 11](#_Toc438152020)

[7.5: Peruvianum group 12](#_Toc438152021)

[7.6: Biogeographic interpretations of the phylogeny 13](#_Toc438152022)

[S1 Text References: 14](#_Toc438152023)

# S1 Text Section 1: Term­inology

## 1.1: Taxonomy

Species names follow the guidelines described in Peralta, Spooner (1) and Grandillo, Chetelat (2). The present study focuses on *Solanum* section *Lycopersicon*, currently comprising 13 species, including both wild and domesticated *S. lycopersicum* (S1 Table). Three accessions from the two species in *Solanum* section *Lycopersicoides* (*S. lycopersicoides* and *S. sitiens*) were used as phylogenetic outgroups. Accession identifiers beginning with “LA” refer to the TGRC database identifiers (http://tgrc.ucdavis.edu); species designations follow the TGRC database information as of May 1, 2015, and may differ from other (even quite recent) previous designations for specific accessions. For clarity of discussion, when referring to a specific accession, the first three letters of species name are used followed by the accession number. For example, *S. pennellii* LA0716 becomes “*pen-*0716.” We abbreviate *S. lycopersicoides* using “*lyd*” to distinguish it from “*lyc*” for *S. lycopersicum*. As recommended by previous taxonomic treatments, the use of “var. *cerasiforme*” is avoided here [1,3], although the TGRC database identifies *S. lycopersicum* LA2933 as falling under this designation.

## 1.2: Species Groups

This transcriptome-wide study (and other previous phylogenies) identified four major species groupings with strong support within *Solanum* section *Lycopersicon*. However, these species groups do not match any previous unified set of subgroup names. Therefore, we have selected a set of group names from several previous taxonomic treatments [1,4,5] that are descriptive of the supported clades, while acknowledging the long history of taxonomy in this group:

- Esculentum group: *S. cheesmaniae*, *S. galapagense, S. lycopersicum*, *S. pimpinellifolium*
- Arcanum group: *S. arcanum*, *S. chmielewskii*, *S. neorickii*
- Peruvianum group: *S. chilense, S. corneliomulleri*, *S. huaylasense*, *S. peruvianum*
- Hirsutum group: *S. habrochaites,* *S. pennellii*

Within the paper, we use the convention of capitalized, non-italicized group names (e.g., Esculentum group) and capitalized, italicized section names (e.g., section *Lycopersicon*). The term “Galápagos endemic species” refers to *S. cheesmaniae* and *S. galapagense*. “Domesticated” accessions are *S. lycopersicum* reference genome (*lyc*-ref) and “M82” (*lyc*-3475) accessions.

# S1 Text Section 2: Sequencing

## 2.1: Sequencing and Read Processing

Sequencing of the TruSeq libraries (180 bp insert) was performed by the Genome Sequencing Facility of the Greehey Children’s Cancer Research Institute at the University of Texas Health Science Center at San Antonio (http://ccri.uthscsa.edu/). TruSeq libraries were linked to a paired-end flow cell containing complimentary adapters. The raw reads were initially cleaned by removing empty reads and low quality sequences.

Prior to mapping and assembly, reads were trimmed using the SHEAR program (http://www.github.com/jbpease/shear), using these settings:

shear.py --fq1 READPAIR1.fq --fq2 READPAIR2.fq --out1 FILTEREDPAIR1.fastq \
--out2 FILTEREDPAIR2.fastq --trimfixed 0:0 --trimqual 20:20 --filterlength 50 \ --trimpattern3 AGATC --trimpolyat 12 --trimambig --filterunpaired \
--filterambig 8 --filterlowinfo 0.5 --barcodes1 BARCODE1 --barcodes2 BARCODE2 --platform TruSeqDualIndex

Five accessions (*chm*-1316, *arc*-2172, ­*lyc*-2933, *lyd­*-2951, *sit*-4116) were run separately on a TruSeq single-indexed process and the option “–-platform TruSeq” was used with only a single barcode.

SHEAR first removed RNA-Seq adapter sequence contamination with Scythe (https://github.com/vsbuffalo/scythe/). Next, bases were trimmed from either end of the read when they were composed of either Phred32 quality score < 20, poly-A or poly-T runs of length ≥ 12 bp, or ambiguous bases (N’s). When an exact match for the start of the adapter prefix “AGATC” was found at the 3′ end, this was also removed. Trimmed reads were subsequently filtered out if they were less than 50bp in length or had 8 or more ambiguous bases. SHEAR also computes a dinucleotide mutual information score for each read to identify highly repetitive or likely erroneous sequences. Reads where the dinucleotide mutual information score was > 0.5 were considered extremely low-complexity and were removed. This step removed highly repetitive and likely non-genomic reads that could cause problems, particularly with the *de novo* assembly stage. If either read from a pair was removed, both were removed to retain full paired-end mapping functionality. Full statistics on read trimming can be found in S1 Data 1.1*.*

## 2.2: Genome Preparation for Mapping:

*S. lycopersicum* reference chromosome FASTA files (version SL2.50) were downloaded from SolGenomics (ftp://ftp.solgenomic.net). The corresponding annotation GFF3 files for both protein-coding gene models and ncRNA were also obtained (v. ITAG2.4; note SGN annotation and chromosome version numbers are not synchronized). The reference chloroplast genome (GenBank #NC_007898.3) and mitochondrial scaffolds v.1.50 (http://mitochondrialgenome.org/) were also obtained. GFF3 gene annotation files were converted with a custom Python script to the Split Junction tabular format used natively by STAR. The genome was prepared for use with the STAR RNA-Seq spliced aligner [6] using the following parameters:

STAR --runMode genomeGenerate --genomeDir ./ --genomeFastaFiles \
SL2.50ch00.fasta SL2.50ch01.fasta SL2.50ch02.fasta SL2.50ch03.fasta \ SL2.50ch04.fasta SL2.50ch05.fasta SL2.50ch06.fasta SL2.50ch07.fasta \ SL2.50ch08.fasta SL2.50ch09.fasta SL2.50ch10.fasta SL2.50ch11.fasta \
SL2.50ch12.fasta SLmt.fasta SLcp.fasta --sjdbFileChrStartEnd \ ITAG2.4_introns.txt --sjdbOverhang 99

## 2.3: RNA-Seq mapping and Alignment Processing:

Reads from all 29 accessions were mapped using STAR RNA-Seq spliced aligner [6] using the following command (default parameters).

STAR --genomeDir SL2.50 --readFilesIn FILTEREDPAIR1.fq FILTEREDPAIR2.fq \
--outFileNamePrefix ACC1--outSAMorder PairedKeepInputOrder \
--outReadsUnmapped Fastx --outSAMtype SAM

The total proportion of uniquely mapped reads was 90.7%. Outgroup accessions were mapped at reduced rates (73-80%).

SAM files produced by STAR were converted to sorted BAM files using SAMtools v. 0.1.19 [7] with the following commands:

samtools view -bS FILE.sam | samtools sort – FILE.sorted

SAMtools *mpileup* was then used to call alleles from the BAM files for all accessions for each chromosome.

samtools mpileup -uD -f SL2.50.fa -r SL2.50ch01 FILE.sorted.bam \ FILE.sorted.bam [...] | bcftools view -cg - > FILE.SL2.50ch01.vcf

VCF files were processed into Multisample Variant Format (MVF) files using *vcf2mvf* from the MVFtools package [8]. Multiple quality cutoffs were used to form three primary datasets:

- Lower-Quality (LQ) alignment that includes all mapped sites regardless of allele call quality. The LQ alignment was used to generate the more high-quality datasets; it was not used for any analyses, but is provided as supporting data.
- High-Quality (HQ) alignment with allele call Phred score ≥ 30 and mapped read coverage ≥ 3. The HQ alignment was used for the majority of phylogenetic tree inference and introgression testing.
- High-depth (HD) alignment with allele call Phred score ≥ 30 and mapped read coverage ≥ 10. The HD alignment was used to estimate rates of heterozygosity and for analyses of shared heterozygosity. The higher coverage requirement allows for more confident determination of the heterozygosity or homozygosity of a given site.

Additionally, two datasets were derived from the HQ dataset for use in phylogenetic inference and introgression testing. The “HQ10” dataset contains only sites with a minimum of 10 accessions represented for each site, and was used for phylogenetic tree inferences. The “HQcomb” dataset was created by combining sequences from the three outgroup accessions. Specifically, the combined outgroup represents the *lyd*-4126 (highest coverage outgroup) with alleles from either *lyd­*-2951 or *sit­*-4116 inserted at sites where *lyd­*-4126 was missing data. The “HQcomb” dataset was used for all *D*-statistic analyses and introgression testing in order to maximize coverage. Unless otherwise noted, all analyses described were conducted using the HQ dataset.

## 2.4: *de novo* assembly of unmapped reads

Reads that did not map to the reference genome for each accession using the STAR RNA-Seq spliced align method above, were separately assembled using Trinity v. 2014-07-14 [9]. 694,699 transcripts (272 Mb) across all 29 accessions were assembled. Of these, 529,881 (76.2%) mapped to the SL2.50 reference genome using GMAP v.2014-05-15 [10]. However, upon closer inspection of the mapped alignments, we detected grossly elevated substitution rates on these mapped transcripts and the clear clustering of substitutions at the terminal ends of the mapped transcripts. We determined that the vast majority of these mapped transcripts were assembled from multiple reads whose overlapping portions were sufficient for a *de novo* assembly match, but whose non-overlapping sections contained significant sequencing errors. We confirmed this in particular using *lyc*-3475 (M82), which is ~ 0.04% divergent in transcribed regions from the tomato reference sequence. Therefore, to prevent introducing error into the main dataset, these transcripts were not integrated.

The remaining 164,818 unmapped *de novo* transcripts (67.9 Mb) were fragmentary (median = 246 bp, N_50_ = 432 bp). A BLAST search of GenBank revealed 74,129 (44.9%) of them were likely from traces of plant-specific microbiota or other minute contaminants. The BLAST results were further used to categorize them into four groups: [1] likely Solanaceae sequences, [2] likely plant sequences, or [3] unknown origin. We clustered these sequences using MCL, but this revealed no significant orthologous groups of non-reference transcripts. Therefore, these sets of *de novo* filtered transcripts were not included in the results of this study, but are available as supplementary data (URL PENDING).

# S1 Text Section 3: Phylogenetic and genetic distance analyses

## 3.1: Whole-transcriptome, chromosome, genomic window and gene phylogenies

Using MVFtools (<http://www.github.com/jbpease/mvftools> [8]), alignments were prepared from the HQ dataset using the following data partitions: (1) transcriptome-wide, (2) each chromosome, (3) ITAG2.4 annotated cDNAs (including UTRs and intronic regions, if sequenced), (4) 100 kb non-overlapping genomic windows, (5) 1 Mb non-overlapping genomic windows. Phylogenies were inferred using several methods (RAxML [11], ASTRAL [12], MP-EST [13]) and partitions of the data. The HQ10 dataset was used to create a transcriptome-wide alignment from 46,566,262 aligned sites (Fig 2A and S2A Fig). In addition, phylogenies were created for subsets of taxa from the full alignment (S2B–C Fig). We inferred phylogenies for each chromosome separately (S2 Table), for 100 kb and 1 Mb genomic windows and for each reference-annotated gene (see S2 Table for summary). For the 100 kb and 1 Mb genomic windows, the unplaced scaffolds labeled as SL2.50ch00 were not used, since they are not spatially ordered. For the whole-transcriptome and chromosome concatenated alignments, a minimum coverage of at least 10 accessions was required at a site for inclusion in the alignment. All concatenated trees were generated using RAxML v. 8.0.26 [11] with the following command:

raxmlHPC-PTHREADS -s ALIGN.phy -n JOBNAME -m GTRGAMMA -p [###] –f a –N 100

Bootstrapping was performed on the whole-transcriptome tree and on the window trees. We performed 100 runs of ASTRAL by bootstrapping the set of 100 kb trees. We also performed 100 bootstrap replicates of 100 kb trees for MP-EST, taking the tree with best likelihood score from a set of 10 MP-EST runs per bootstrap replicate. For the whole-transcriptome tree shown in Fig 2 and chromosomal trees (S2 Table), *S. huaylasense* LA1360 was excluded since our data (S4A–B Fig) agree with previous findings that it represents an introgressed/hybrid lineage [14] (see also main text). To further support our decision to omit this taxon from the consensus tree, we computed the taxonomic instability index (TII) [15], as implemented in RogueNaRok [16], for all taxa including *hua*-1360, using trees containing no polytomies and all 30 taxa from both individual genes and trees from 100 kb windows (see S1 Data 1.26). This ‘rogue taxon analysis’ indicates that *hua*-1360 has the highest TII values of any accession; for a given taxon, high TII indices indicate its position in the tree topology relative to other taxa is relatively inconsistent, while low indices indicate taxa with more consistent topological placement.

A phylogeny with all accessions (including *hua-*1360) and other subsets of accessions were used to compute additional trees (S2A–C Fig).­­ In S2E-F Fig showing coalescent trees, values next to each node represent the number of bootstrap replicate trees that include that node; unlabeled nodes are supported by all replicates (i.e., have 100% bootstrap support).

From 802 Mb of assembled genome sequence, 8000 windows of 100 kb and 800 windows of 1 Mb are possible. Despite the large amount of gene-poor heterochromatin in the tomato genome, alignment data from RNA-Seq mapping was available for 4545 regions of 100 kb and 640 regions of 1 Mb. Out of 4545 100 kb trees, 2642 (58%) had at least 1000 sites aligned for all 29 accessions (max = 58193 sites, min = 1694 sites). 100 kb windows were sampled from both arms on all 12 chromosomes (except for chromosome 2, which is acrocentric). Using these phylogenies inferred from the data at various scales, in the main text and below we discuss several features of the phylogeny of *Solanum* section *Lycopersicon* as a whole and within the major subgroups, including our findings’ agreement with previous population surveys and phylogenies (S1 Text Sections 7.1–7.5).

A “cloudogram” [17] of 100 kb window trees (Fig 2B) was prepared using DensiTree v 2.2.1 [18]. 100 kb Trees were rooted and converted to ultrametric using *chronopl* function from the *ape* R library (<http://cran.r-project.org> [19]); default settings were used with lambda=0.

To exclude homoplasy as an explanation of the phylogenetic discordance observed among loci and windows, we plotted gene tree discordance against internode length and found that shorter internodes do exhibit more conflict, similar to the findings of [20]. This analysis suggests that homoplasy can be excluded as a major contributor to observed discordance, although it is unclear whether this analysis can distinguish the effect of introgression from ILS. Both of these processes are more likely to act when there are short internal branches.

## 3.2: Heterozygosity and Pairwise *d*_N_ and *d*_S_

Using the HD alignment, genome-wide levels of heterozygosity were calculated for each accession (S1 Data 1.3). All our transcripts in this analysis were reference-based mapped, excluding all but very recent duplicates from our above analyses. However very recent gene duplicates that gave rise to closely related paralogs could influence our analyses by producing elevated estimates of heterozygosity at these recently duplicated loci. In order to verify that our heterozygosity estimates were not driven by gene duplicates, we also computed heterozygosity for a set of single-copy genes for which we could identify single-copy orthologs in *Arabidopsis thaliana*. A list of *Arabidopsis thaliana* gene IDs for 357 ultra-conserved orthologs (UCOs) was obtained from the *Compositae* Genome Project (http://compgenomics.ucdavis.edu). Orthologous genes in the *S. lycopersicum* reference gene set were obtained by reciprocal best BLAST hit in the annotated ITAG2.4 reference genes. Of the alignments of these 357 genes, 312 genes had 100 or more nucleotide sites from which to estimate heterozygosity. Levels of heterozygosity in UCOs are lower (as expected for a highly conserved gene set), but were proportionate to the HD and genome wide estimates, indicating that pervasive gene duplication does not explain estimates of heterozygosity from our RNA-Seq data. More specifically, it shows that a particular lineage was not affected by an increased rate of gene duplication that would cause incorrect mapping and confound our estimates of within accession heterozygosity.

For completeness, we estimated transcriptome-wide *d*_N_ and *d*_S_ values between all species pairs (S1 Data 1.4) using standard methods. Using PAML v.4.8a [21], these *d*_N_ and *d*_S_ pairwise values were calculated from the HQ alignment using ITAG v.2.4 reference annotated gene boundaries.

## 3.3: Divergence times

To estimate dates of divergence (Fig 2A), we used *r8s* [22] to infer an ultrametric, molecular clock tree from the RAxML transcriptome-wide concatenated phylogeny using the Penalized Likelihood method. Times were calibrated using two fixed date estimates. First, the divergence of *S. sitiens* and *S. lycopersicoides* was fixed at 1.81 Ma [23]. Second, because there are no reliable estimates of substitution rate or generations-per-year in this group, we used the estimated time of divergence (8 Ma; [23]) and percent sequence divergence (8.7%; [24]) between potato and tomato to calibrate the timing of divergence of the self incompatible lineages in the Hirsutum group (*hab*-1777, and *pen*-3778) from all other non-Hirsutum group, non-domesticated accessions of tomato species. Using these numbers, we then estimated the divergence of the ancestral *S. pennellii* and *S. habrochaites* lineage from the remainder of section *Lycopersicon* at 2.48 Ma.

Estimated divergence times (Fig 2A) indicate that sections *Lycopersicon* and *Lycopersicoides* separated approximately 4.7 million years ago (Ma). Within *Lycopersicon*, the divergence of the Hirsutum group from the others is estimated at 2.48 Ma, followed closely by the Peruvianum group’s separation from the Esculentum + Arcanum lineage at approximately 1.89 Ma. The Esculentum and Arcanum groups are estimated to split at approximately 1.44 Ma. Therefore, we estimate that all four of the major groups of wild tomato were established within a short span of ~1 million years.

## 3.4: Chloroplast and mitochondrial sequences

In addition to the nuclear genome, all RNA-Seq reads sets were mapped to the chloroplast genome (NCBI #NC_007898.3), and to the mitochondrial scaffolds. However, insertion of chloroplast and mitochondrial sequences in the nuclear genome are very common and dispersed throughout the tomato genome [24]. A BLAST search of the SL2.50 tomato reference genome revealed many insertions of sequences from both organelles, including ones of up to 65 kb (40%) of the chloroplast genome and up to 21 kb of the mitochondrial scaffolds (S1 Data 1.7). Given low sequence divergence and with only transcriptome data, we were unable to confidently place many of the RNA-Seq reads specifically in the organelle genomes instead of their nuclear copies. Therefore, organelle genomes were not included in the phylogenetic reconstruction.

# S1 Text Section 4: Introgression analyses

As expected from the recent divergence and complex history of this clade, we observed a wide range of genome-wide *D*-statistics, and large variation among lineages in the frequency of inferred introgression (S1 Data 1.5). Phylogenies indicated that *hua*-1360, *hua*-1364, and *per*-2744 are reticulate for alleles from Esculentum and Peruvianum groups (see S1 Text Section 7.5). This was confirmed in the *D-*statistics; nearly all comparisons involving these accessions exhibited high and significant *D* values. We also observed overall significant genome-wide *D*-values both for *S. habrochaites* accessions relative to *S. pennellii,* and for *S. peruvianum/corneliomulleri* accessions relative to *S. chilense,* compared to all accessions in the Arcanum and Esculentum groups. These observations are indicative of the ancestral introgressions described previously.

In addition to the genome-wide estimates, the window-based *D*-statistics revealed at least four examples of localized chromosomal introgressions between individual accessions. The most striking examples, involving two different *S. neorickii* accessions with red-fruited (likely *S. pimpinellifolium*) donors, are discussed in the main text (Fig 4A). Other localized putative introgressions involve gene exchange between S. *pimpinellifolium* and *S. pennellii,* and between *S. chilense* and *S. pennellii* (S5B–C Fig). These window-based *D*-statistic analyses also highlight the extensive introgression already noted (above) between *cor*-0107 and *hua­*-1358, especially a large region on the long arm of chromosome 1 of ~ 5 Mb (S5D Fig). Nonetheless, the overall scarcity of these small recent introgressions suggests that although species in this group can be readily hybridized artificially, introgression among wild tomato species *in situ* might be quite rare.

# S1 Text Section 5: Lineage-specific evolution analyses

The high levels of incompletely sorted ancestral variation and variability of the gene-by-gene phylogenies present a particular challenge in identifying lineage- or species-specific substitutions. We initially used a more conservative variant of a *d*_N_/*d*_S_ test to infer which genes show high relative frequencies of nonsynonymous substitutions (and therefore are likely under positive selection) for the four well-supported subclades within *Lycopersicon* (Esculentum, Arcanum, Peruvianum, and Hirsutum groups), as well as some specific species. For the four major groups (Esculentum, Arcanum, Peruvianum and Hirsutum) we evaluated evidence for positive selection using the branch-site test in PAML4.8a [21] (see main text Materials and Methods).

We tested for changes on the branch separating section *Lycopersicon vs.* section *Lycopersicoides* and for all other samples against these groups/species: Esculentum group, Arcanum group, Peruvianum group, Hirsutum group, the Galápagos species, *S. pennellii*, *S. habrochaites*, *S. chilense*, *S. chmielewskii*, and *S. neorickii* (S1 Data 1.8–1.20).

Phylogenies for each 14-species gene alignment were then inferred using RAxML v.8.1.16 [11] using standard parameters and the GTR model. For each of the four major groups (Esculentum, Arcanum, Peruvianum, Hirsutum), we verified in the gene tree that all accessions in the given group are a monophyletic clade (i.e., that the gene tree has an appropriate branch ancestral to the group being tested). If this ancestral branch was not present, the gene was not tested for that particular group. Otherwise, the ancestral branch was marked as the target “foreground” branch and tested using the branch-site test in PAML. The following control file parameters were used for the null test:

The following control file parameters were used for the null test in PAML 4.8a [21]:

runmode = 0

seqtype = 1

CodonFreq = 2

model = 2

NSsites = 2

icode = 0

Mgene = 0

fix_kappa = 0

kappa = 2

fix_omega = 1

omega = 0

fix_alpha = 1

alpha = 0.

ncatG = 8

fix_blength = 2

The alternative test uses the same parameters except fix_omega = 0 and omega = 1. We ran both null and alternative tests, and recorded *d*_N_/*d*_S_ values and likelihood scores. Since branch lengths were fixed at the values provided by the RAxML tree, the null model has 4 free parameters and the alternative test has 5. Therefore significance was assessed by a likelihood ratio test (LRT) assuming a *χ*^2^ distribution with 1 degree of freedom.

We estimated the overall proportion of genes undergoing lineage-specific adaptation in two different sets of data, calculating separate false discovery rates (FDR) for each set. First, we looked at the proportion of genes from the genome-wide scan with at least one unambiguous nonsynonymous difference between the target lineage and the non-target accessions. For each target group, we counted the number of total genes that were testable by PAML and the proportion therein with LRT *P* < 0.01. These values are reported in the main text:

Esculentum: 3.08% of genes (137 out of 4447 testable genes; FDR=32.5%),

Arcanum: 4.69% of genes (179 out of 3819 genes; FDR=21.3%)

Hirsutum: 3.97% of genes (38 out of 958 genes; FDR 25.2%)

Second, we tested how many total genes with *d*_N_/*d*_S_  > 1 at *P* < 0.01 were detected by PAML among genes with a gene tree containing the target ancestral branch, regardless of the results of the conservative non-overlapping nonsynonymous allele test described above. Among these genes we found the following rates:

Esculentum: 1.35% of genes (174 of 12854 testable genes; 74.1% FDR)

Arcanum: 5.16% of genes (620 out of 12006 genes; 19.3% FDR)

Hirsutum: 1.65% of genes (56 out of 3390 genes; 60.5% FDR)

# S1 Text Section 6: PhyloGWAS analyses

The following four ecological variables were used to test for association of specific alleles among wild tomato accessions with environmental conditions:

1. Altitude/temperature: 23 accessions total were tested (16 from 0–689 meters above sea level; 7 from 2147–3540 m.a.s.l.). Altitude was found to have a strong inverse correlation with mean annual temperature (*r* = −0.88), therefore these variables were combined into a single ecological axis. Low altitude accessions are *che*-3124, *che*-0429, *chi*-1782, *cor*-0107, *cor*-0444, *gal*-0436, *gal*-3909, *hab*-0407, *hua*-1358, *lyc*-2933, *pen*-0716, *pen*-3778, *per*-2744, *per*-2964, *pim*-1269, and *pim*-1589. High altitude accessions are: *arc*-2172, *chi*-4117, *chm*-1028, *chm*-1316, *hab*-1777, *neo*-1322, and *neo*-2133.
2. Latitude/climate seasonality: 19 accessions total were tested with 8 accessions sampled from −1.3° to −11.5° latitude coastal zone and −13.3° to −13.8 on the Eastern Cordillera, and 11 accessions sampled from −13.0° to −22.9° along the coastal zone. We found that latitude correlates inversely with annual mean PAR (*r* = –0.85), month variation in PAR (s.d. of monthly mean PAR, *r* = –0.82), temperature annual range (*r* = −0.81), and to a lesser degree with annual precipitation (*r* = 0.55). We designated the more northern climate zone “constant,” including the accessions: *arc*-2172, *chm­*-1028, *chm*-1316, *hab*-0407, *hab*-1777, *hua­*-1358, lyc-2933, *neo*-1322, *neo*-1322, *pim*-1269, and *pim*-1589. Accessions from the more southerly form were designated climatically “variable,” including *chi*-4117, *chi*-1782, *cor*-0107, *cor*-0444, *pen*-0716, *pen*-3778, *per*-2744, and *per*-2964.
3. Interpolated water pH: 9 accessions total were tested (4 from “acidic” environments (pH ≤ 6); 5 from “basic environments (pH ≥ 7.5). Acidic accessions are *che­*-0429, *gal*-0436, *lyc*-2933, and *neo*-1322. Basic accessions are *pim*-1589, *chi*-1782, *per*-2744, *per*-2964, and *chi*-4117.
4. Heavy metals sampled from sediment (As, Cu, Hg, Ni, Pb): 9 accessions total were tested, with 4 accessions from “low metal” environments and 5 from “high metal” environments. High metal accessions are *chi*-1782, *cor*-0107, *cor*-0444, *pen*-0716, and *pim*-1269. Low metal accessions are: *chm*-1028, *chm*-1316, *neo*-1322, and *hab*-1777.

To assess whether the number of allelic differences found to be segregated exclusively between our environmentally defined populations was greater than expected by chance, we used a simulation approach. To generate the null distributions we simulated loci with one variant site over the consensus species tree using the program ms [25]:

ms 58 1000000000 -s 1 \
-I 29 2 2 2 2 2 2 2 2 2 2 2 2 2 2 2 2 2 2 2 2 2 2 2 2 2 2 2 2 2 \
-ej 0.04932 27 26 -ej 0.07053 29 28 -ej 0.08274 26 25 \
-ej 0.16611 23 22 -ej 0.17418 20 19 -ej 0.20942 25 24 -ej 0.22874 3 2 \
-ej 0.29367 22 21 -ej 0.30337 18 17 -ej 0.35478 28 24 -ej 0.45527 24 21\ -ej 0.51688 17 16 -ej 0.70747 7 6 -ej 0.73152 19 16 -ej 0.76898 5 4 \
-ej 0.98585 13 12 -ej 0.99785 15 14 -ej 1.00183 11 10 -ej 1.1448 12 10 \
-ej 1.18711 9 8 -ej 1.33277 14 10 -ej 1.5878 21 16 -ej 1.61987 10 8 \
-ej 1.81 2 1 -ej 1.94077 6 4 -ej 1.99408 16 8 -ej 2.26851 8 4 \
-ej 4.42059 4 1

For the ms simulation, coalescence times were taken from the ultrametric consensus tree (Fig 2A).  A single variable site ("-s 1") for 29 diploid individuals ("58" = 2 x 29) for one billion replicates was simulated over these coalescent times. Each coalescent unit is 1 Myr since 4*N_e_* = 4 × 10^5^ individuals × 2.5 generations per year). Individual simulations were randomly grouped into datasets of the appropriate size for each of the four contrasts. We then examined the allele patterns for each simulation run to determine whether the single site was perfectly associated with the groups of accessions distinguished by our environmental axes. Note that our number of simulated datasets was limited by the computational time required to simulate each tree but, given the distribution of simulated values for three of our factors, the *P*-values are likely to be much lower than estimated.

For the altitude/temperature test (Factor 1), no gene showed a nonsynonymous difference out of a total of 233,567 variable amino acid sites. For 4214 replicate sets of 233,567 simulated genes, an average of 0.03 (range 0–2) simulated allele patterns per replicate matched the PhyloGWAS pattern. Of all our PhyloGWAS tests, this one required the most complex allele pattern, including 23 accessions and 2 pairs of conspecific accessions split between the two ecological groupings. Therefore, this specific test might indicate an upper limit to this kind of association analysis, or simply that adaptation to this stressor did not occur via selection on coding changes found among ancestral variation.

For the latitude-seasonality test (Factor 2), 21 genes with nonsynonymous differences were observed out of a total of 253,161 variable amino acid sites. For 3949 replicate sets of 253,161 simulated genes, an average of 0.002 (range 0-1) simulated allele patterns per replicate matched the PhyloGWAS pattern for this test; in other words, none of the simulated datasets had 21 or more genes perfectly associated with the environmental variables. This makes our *P*-value *<* 2.53×10^−4^ (i.e., less than 1/3949).

For the pH test (Factor 3), 43 genes were observed with nonsynonymous differences out of a total of 160,255 aligned sites with variable amino acids. For 6323 replicate sets of 160,255 simulated genes, an average of 5.28 (range 0–15) simulated allele patterns matching the tested PhyloGWAS pattern (*P* < 1.58×10^−4^). We noted that several loci with nonsynonymous differences were transferases, oxidases, and other catalytic enzymes that might be affected by or respond to soil pH. In addition, several loci were associated with plant disease resistance responses, including antifungal responses (e.g. *Solyc02g082920*). Soil pH can be important for determining the presence and abundance of soil borne diseases, so these variants might reflect different selective conditions for disease resistance for soil borne pathogens. Only *Solyc05g005460* (*nucleoredoxin 2*) had 2 nonsynonymous differences between the two ecological groupings.

For the metal test (Factor 4), 404 genes were observed with nonsynonymous differences between environments with higher versus lower presence of heavy metals, out of a total of 198,908 variable amino acid sites. For 5023 replicate sets of 198,908 simulated genes, an average of 25.96 (range 10–43) simulated allele patterns matched the PhyloGWAS pattern. This gives our observed value a *P*-value of *P* < 1.99×10^4^. Gene lists from all four tests are available in S1 Data 1.22−1.25.

# S1 Text Section 7: Phylogeny of *Solanum* section *Lycopersicon* and major groupings

## 7.1: Sections Lycopersicon and Lycopersicoides

Sections *Lycopersicoides* and *Lycopersicon* were found to be monophyletic clades with high support in all datasets. *lyd*-2951 and *lyd*-4126 were inferred as sister in all phylogenies with ~99% of window and gene tree support, with *sit*-4116 inferred as sister to both with equally high support. The clear separation in % sequence divergence and strong support for this split between the two sections validate the use of section *Lycopersicoides* as an outgroup lineage. Whole-transcriptome and subsection phylogenies indicate the presence of four major groups as suggested by other recent clade-wide molecular studies [1,26]. The Hirsutum group (*S. habrochaites*, *S. pennellii*) is inferred to diverge first from the rest of the section, followed by the Peruvianum group (*S. chilense, S. corneliomulleri*, *S. huaylasense*, *S. peruvianum*). The Esculentum (*S. cheesmaniae*, *S. galapagense, S. lycopersicum*, *S. pimpinellifolium*) and Arcanum groups (*S. arcanum*, *S. chmielewskii*, *S. neorickii*) are inferred with strong support as sister groups (Fig 2 and S2 Fig). While support for these groupings is generally strong among window-based phylogenies (S2 Table), the high levels of incompletely sorted ancestral alleles (S2 Fig) and indications of ancestral introgressions (S4E–F Fig and below) lead to some uncertainty in the relationships of species within and among these groups.

## 7.2: Hirsutum group

Within section *Lycopersicon*, the Hirsutum group is inferred to be monophyletic and sister to the rest of the clade with high bipartition support. The pairs of accessions for *S. pennellii* and *S. habrochaites* accessions are each inferred to be monophyletic in 96-99% of 100 kb and 1 Mb phylogenies and on all 12 chromosomes. However, even though *S. pennellii* and *S. habrochaites* are inferred as sister on all 12 chromosomes, only 60% of 100 kb windows and 75% of 1 Mb windows support these two species as sister taxa. Among genes, strict monophyly of all four *S. habrochaites* and *S. pennellii* accessions is only observed in 35% of genes, though in general gene-by-gene trees are more variable in these smaller partitions given the high level of ILS.

To investigate these patterns of gene tree discordance, we inferred trees from all reference genes using only *pen*-3778, *hab*-1777, *pim*-1589, and outgroup *lyd*-4126. Out of 20159 gene trees inferred for these three accessions, we observed these proportions of gene tree topologies: (*hab*,*pen*)*pim* = 8924 (44.3%), (*hab*,*pim*)*pen* = 6482 (32.2%), and (*pen*,*pim*)*hab* = 4753 (23.5%). Nearly identical gene tree proportions are found when *neo*-1322 from the Arcanum group is used in place of *pim*-1589, and with any combination of *S. habrochaites* and *S. pennellii* accessions. This imbalance in discordant gene trees and the consistency of this pattern using different combinations of accessions together indicate introgression occurred among ancestral populations. Specifically, we infer that introgression occurred between the *S. habrochaites* ancestral population (prior to the divergence of the populations from which *hab*-1777 and *hab­*-0407 were sampled), and an ancestral Esculentum/Arcanum lineage prior to (or very close in time to) speciation event within these two groups; this inference is supported by *D*-statistic analyses (below). From the imbalance in gene tree proportions, we estimate that approximately 32.2% – 23.5% = 8.7% of genes are affected by this ancestral introgression (S4E Fig).

The uncertainty in the relationship of *S. pennellii* and *S. habrochaites* is reflected in previous molecular phylogenies, which most often place them as sister species [26,27] though frequently with low bipartition or bootstrap support or as a polytomy [28,29]. *S. pennellii* and *S. habrochaites* are also distinct ecologically (*S. pennellii* is generally coastal, *S. habrochaites* is generally montane; S1 Fig). Phylogenies based on morphological or chromosomal inversion data are more likely to place *S. pennellii* as diverging from the rest of the section before *S. habrochaites* [30,31], consistent with karyotype investigations that indicate small inversions differentiating *S. pennellii* and *S. habrochaites* [32]. However, phylogenetic inference based on inversions alone is inconsistent with the transcriptome-wide molecular analysis.

## 7.3: Esculentum group

The Esculentum group is composed entirely of self-compatible species, including wild and domesticated *S. lycopersicum*, its closest wild relative *S. pimpinellifolium*, and the two Galápagos endemic species, *S. cheesmaniae* and *S. galapagense* [33]. The group is highly supported on all 12 chromosomes and in 98– 99.4% of phylogenies inferred from genomic window and gene trees. However, considerable variability in the phylogenetic relationships within this group was observed in the window-based and chromosomal trees. This is consistent with previous studies that have shown variable phylogenetic placement of species within the group, possibly indicative of interbreeding and introgression among these species [3,34-40] as well as their very recent and rapid evolutionary divergence.

Within the group, we infer two subclades. The reference genome and domesticated *lyc­*-3475 (a.k.a. the “M82” cultivar) were found to be more closely related to *S. cheesmaniae* and *S. galapagense*, while *lyc*-2933 was inferred to be more closely related to *S. pimpinellifolium*. This is consistent with evidence from population studies that have shown that wild *S. lycopersicum* accessions sampled from the same geographical region as *lyc*-2933 are enriched for *S. pimpinellifolium*-like population markers [36,40]. The whole-chromosome phylogenies for chromosomes 1, 4, and 9 indicate monophyly of *pim*-1589 and *lyc*-2933 to the exclusion of *pim*-1269, indicating additional evidence of introgression/interbreeding between *S. pimpinellifolium* and *S. lycopersicum* wild populations.

The sister relationship of the Galápagos species is supported only moderately (55.3% of 100 kb trees) as is the monophyly of the two *S. galapagense* accessions (59% of 100 kb trees). The two accessions of *S. cheesmaniae* are not inferred as sister in the whole-transcriptome consensus, and are weakly supported as sister in 24% of 100 kb trees. This result is consistent with previous findings that *che*-3124 has a closer relationship with domesticated *S. lycopersicum* than to other *S. cheesmaniae* and *S. galapagense* accessions [41], possibly because of introgression from this wild species into domesticated lineages. Therefore, the Esculentum group is found to be a group with clear phylogenetic distinction from the rest of the clade, but with complex genetic relationships within the clade that emphasize their very recent divergence, and the importance of biogeographic structure and ongoing interbreeding, especially directed introgression into the domesticated lineages.

## 7.4: Arcanum group

The Arcanum group is composed of three species (*S. arcanum*, *S. chmielewskii*, *S. neorickii*) that are generally found at high altitudes and vary in the presence of genetic self-incompatibility. *S. neorickii* is completely self-compatible and likely frequently selfing, while *S. chmielewskii* exhibits higher genetic variability and perhaps evidence of more frequent facultative outcrossing; *S. arcanum* populations are generally self-incompatible but with some mixed self-incompatible/self-compatible and facultatively self-compatible accessions [1,42-44]. Geographically, *S. chmielewskii* is found in a small region in central Eastern Cordillera of the Andes (S1 Fig). *S. neorickii* is also found in the Eastern Cordillera range, but over a much broader range, stretching up to the southernmost end of the Northern Andean range in Ecuador. *S. arcanum* was until recently referred to as *S. peruvianum*, though many previous studies had noted its genetic dissimilarity from the rest of *S. peruvianum* populations and referred to it variously as *S. peruvianum* var. *humifusum* or “North” [1,28]. Geographically, *S. arcanum* presently occupies a relatively small area centered on the Amotape-Huancabamba zone (S1 Fig), in the valleys of Río Huancabamba and Río Marañón, spanning both the Central and Eastern Cordilleras. Genetic and morphological variability in *S. arcanum* is known to be high and four types of *S. arcanum* have been previously distinguished [45].

The Arcanum group is supported strongly by all 12 chromosomes and in 95–98% of window trees. *S. chmielewskii* accessions are inferred to be sister with very high bipartition support (95−99% of window trees). *S. neorickii* support is somewhat lower (75−95% of window trees), in part due to a variable relationship with *arc*-2172. The two *S. neorickii* accessions are strongly supported as monophyletic relative to *S. chmielewskii* even though they are from the opposite ends of *S. neorickii*’s range; there is little evidence of the southern *neo*-1322 preferentially grouping with *S. chmielewskii* in window or gene phylogenies.

As indicated by the high support for the Arcanum group in all phylogenies, *arc*-2172 is supported strongly as sister to *S. neorickii* with no clear indications of introgression or interbreeding with Peruvianum group accessions. The placement of *S. arcanum* within the Arcanum group is also well supported by Labate, Robertson (14) that show clear relationships between these three species, except in the case of a few *S. arcanum*  accessions, one of which was recently reclassified as a *S. huaylasense* (see discussion on *hua­*-1360 below).

## 7.5: Peruvianum group

The Peruvianum group exhibits more complex phylogenetic relationships than the other subgroups. This group (a.k.a. the Peruvianum complex) historically was labelled as two species *L. peruvianum* and *L. chilense* (when the genus name was *Lycopersicon*), with *S. corneliomulleri* (J.F. Macbr.), and *S. huaylasense* and *S. arcanum* recently distinguished from this species complex [45]. The Peruvianum group itself is supported in the whole-transcriptome phylogenies (Fig 2 and S2 Fig) and all 12 chromosomes (only when *hua*-1360 is excluded, see below). Support for the group is low in window-based phylogenies with only 33% of 100 kb trees and 57% of 1 Mb trees supporting monophyly of all Peruvianum group accessions. No species within this group except *S. chilense* is consistently supported as monophyletic from window or gene trees.

Even though they are from two geographically distinct locations, our two *S. chilense* accessions group as sister in 63-89% of window trees, depending on the size of the window. They also appear as distinct from the rest of the Peruvianum group in the Peruvianum group tree (S2C Fig). A recent study of crossing in wild tomato species showed no apparent post-mating prezygotic reproductive barriers between *S. chilense* and the rest of the Peruvianum group [42].

*S. peruvianum* and *S. corneliomulleri* were poorly supported as separate species in the consensus phylogeny and window phylogenies (5-59%). This result is consistent with many population surveys (S1 Fig), phylogenies, and breeding experiments that show little evidence of genetic or reproductive isolation among populations falling under these two taxonomic designations [1,27,42,46,47]. Given that the two pairs of accessions of *S. corneliomulleri* and *S. peruvianum* are geographically closer to their conspecific accessions (Fig 1), geographic structure is possibly sufficient to explain what little phylogenetic evidence supports the genetic separation of these two species.

We sampled three accessions of *S. huaylasense*, all of which showed highly discordant and variable relationships to other lineages within section *Lycopersicon*. Consistent with Labate, Robertson (14), *hua*-1360 appears to be an introgressed/hybrid lineage (LA1360 is #7 in their study) and other accessions of *S. huaylasense* show considerable evidence of gene flow with Arcanum group or Esculentum group species. Based on gene tree relationships (S4A–B Fig), we infer that *hua*-1360 has been introgressed (perhaps relatively recently) by a species from the Esculentum group. This is consistent with the original collection notes in the TGRC database (<http://tgrc.ucdavis.edu>) for this accession that records the presence of *S. pimpinellifolium* and *S. habrochaites* in the same habitat, including in such close proximity that their branches were physically intermingled. The introgression of Esculentum group alleles into *hua*-1360 has a dramatic effect on the whole-transcriptome concatenated species tree when this accession is included (S2A Fig and S2C Fig compared to Fig 2, S2B Fig, and S2D–F Fig).

From the consensus phylogeny and gene trees, we also infer that *hua*-1364 is either related to *hua­*-1360 and/or independently introgressed by an Esculentum/Arcanum population, though to a lesser degree than in the preceding case (S4C Fig). The Chilean accession *per*-2744 also appears to have a marginally closer relationship to the Esculentum/Arcanum lineage or to the two *S. huaylasense* accessions (or both) than other *S. corneliomulleri* and *S. peruvianum* accessions. The close relationship may be the result of older introgression and/or geographic population structure, since previous surveys that have shown evidence that population markers generally associated with more northern *S. peruvianum* accessions are also found in the isolated habitat region of Chile where *per*­-2744 was sampled [46,48].

Unlike the other two *S. huaylasense* accessions, *hua­_­_*_­_*_­­_*-1358 does not show evidence of hybridization with species outside the Peruvianum group but does have a close relationship with *S. corneliomulleri* (particularly *cor*-0107), likely as the result of recent introgression. Large, spatially localized sequence regions of strong phylogenetic support and reduced pairwise sequence distance with *cor*-0107 are observed on chromosomes 1, 2, 4, 5, and 9 (S5D Fig). These patterns are reflected in the phylogenies of these chromosomes (S2 Table), where the two *S. corneliomulleri* accessions are not sister. Accordingly, introgression appears to be a factor that distorts the relationships of all three *S. huaylasense* accessions used in this study, and may indicate a broader pattern of admixture specifically for other accessions that have been labelled as *S. huaylasense*, as indicated by Labate, Robertson (14). The observation of apparently pervasive introgression/hybridization involving *S. huaylasense* is consistent with the uncertain phylogenetic status of this species, its limited geographic range (collected from only three valleys in Peru, S1 Fig), and the frequent observation of high phenotypic variability in the collection notes (<http://tgrc.ucdavis.edu>).

Similar to the analysis using *S. habrochaites,* we tested for ancestral introgression between the species in the Peruvianum group and other major subgroups of wild tomatoes, using *chi­*-4117A and *cor*-0107 as representative accessions. We observed that the *S. peruvianum* + *corneliomulleri* ancestral lineage shows signs of ancient introgression with the Esculentum group in ~8.8% of genes, relative to *S. chilense* (S4F Fig and S1 Data 1.6). Finally, we wanted to assess whether the observed introgressions and reticulations involving members of the Peruvianum group were affecting the inferred relationships among the major subgroups. When the three accessions that phylogenetically group with the Esculentum and Arcanum lineages are removed and the phylogeny is recomputed (S2B Fig), the Peruvianum group still appears as sister to the Esculentum and Arcanum groups, demonstrating that the group relationships in the consensus tree are not driven by gene flow involving the three specific accessions that have unusually high signatures of discordance/introgression.

## 7.6: Biogeographic interpretations of the phylogeny

Using our consensus phylogeny and the inferred regions of possible ancestral and recent introgression, in conjunction with current species ranges (S1 Fig) and historical/geological information about this region, we can hypothesize a tentative model for the historical geographic spread and diversification of lineages of wild tomato (S6 Fig). The Peruvianum group shows high levels of ancestral variation and sympatry with *S. sitiens* and *S. lycopersicoides* in the Atacama region of northern Chile. Recent evidence suggests that this region has been desert since at least 14 Ma and possibly continuously since 150 Ma [49,50]. These two factors indicate that the Peruvianum group likely is the extant lineage with the most similarity to the ancestral *Lycopersicon* population.

*S. pennellii* and *S. habrochaites* are found in sympatry with each other and with Peruvianum species. However, *S. pennellii* is generally restricted to the coastal zone below altitudes of 1500m, while southern populations of *S. habrochaites* are almost exclusively found inland at higher altitudes. Our data suggests ancestral introgression between the Arcanum/Esculentum ancestral lineage and *S. habrochaites* (prior to the split of the two populations we sampled); this implies that *S. pennellii* and *S. habrochaites* likely diverged nearer to the northern end of their range, as this places *S. habrochaites* in earlier (more ancient) contact with Arcanum/Esculentum lineage populations. This is supported by evidence of population structure in *S. habrochaites* and *S. pennellii* that is centered on the Amotape-Huancabamba zone (AHZ), although other interpretations of the genetic variability in this region have been proposed [51,52].

We also infer that the ancestral Arcanum + Esculentum lineage likely split from the Peruvianum group near the AHZ. This is supported by the present range distributions of members of this group, including *S. arcanum*—which has only been observed around the AHZ region—and *S. pimpinellifolium*—whose range is approximately centered on the AHZ. The Arcanum group and *S. lycopersicum* are the only species observed east of the Andean ridge on the Eastern Cordillera and in the Amazon basin. Given the current distribution of the three Arcanum group accessions and the topography of the region, we hypothesize that the members of the Arcanum group dispersed and diversified via eastern migration of their ancestral (*S. arcanum-*like) lineage through the Huancabamba depression along the Río Marañón valley. Subsequently, *S. neorickii* and *S. chmielewskii* expanded their ranges north and south along the foothills of the Eastern Cordillera to their present ranges. This migration via the AHZ region appears to be the most likely route even though the current southern *S. chmielewskii* and *S. neorickii* are geographically closer to current southern populations of *S. habrochaites* and to the Peruvianum group; the Andean range between these groups is mostly >4000m in altitude—higher than where any modern tomato population has been observed—therefore this section of the Andes likely presented a considerable (likely unpassable) biogeographical barrier.

Given the present distribution of mainland *S. pimpinellifolium* and wild *S. lycopersicum* populations, it is plausible that the Esculentum group also diverged from the Arcanum group in the AHZ. From this central, location *S. pimpinellifolium* likely spread north and south along the coastal region and east along the Río Marañón (though not as far as Arcanum group species). The presence of *S. cheesmaniae* and *S. galapagense* only on the Galápagos Islands, indicates the endemic species diverged on the islands likely from an ancestral *S. pimpinellifolium* or *S. lycopersicum* migrant from mainland Ecuador, as hypothesized by Darwin, Knapp (33). The currently reported accessions of *S. pimpinellifolium* and *S. lycopersicum* found on the Galápagos are likely to be more recent introductions via humans, although we cannot exclude more ancestral associations with the endemic species without sampling these specific populations.

This general south-to-north trajectory mirrors many other animal and plant lineages that appear to have spread from the Atacama region up the Peruvianum coastal desert into Ecuador [53-58]. Based on geological and climatological evidence, this general migration pattern may have been in response to increased desertification of the Peruvian coast. This drying is hypothesized to be due to dry air from the Humboldt/Peru current, reinforced by the rain shadow created by the Andean uplift [59-62]. This period also overlaps or closely follows a global climate shift that appears to have expanded dry grasslands worldwide [63,64], including in the Andean region and Argentina. Therefore, we also see support from other phylogenetic groups and climate models for a general south-to-north migration pattern of wild tomato species.

# S1 Text References:

1. Peralta IE, Spooner DM, Knapp S. Taxonomy of wild tomatoes and their relatives (*Solanum* sect. *Lycopersicoides*, sect. *Juglandifolia*, sect. *Lycopersicon*; Solanaceae). Syst Bot Monogr. 2008;84:1–186.

2. Grandillo S, Chetelat R, Knapp S, Spooner D, Peralta I, Cammareri M, et al. *Solanum* sect. *Lycopersicon*. In: Kole C, editor. Wild Crop Relatives: Genomic and Breeding Resources: Springer Berlin Heidelberg; 2011. p. 129–215.

3. Ranc N, Munos S, Santoni S, Causse M. A clarified position for *Solanum* *lycopersicum* var. *cerasiforme* in the evolutionary history of tomatoes (Solanaceae). BMC Plant Biol. 2008;8(1):130. doi: 10.1186/1471-2229-8-130.

4. Marshall JA, Knapp S, Davey MR, Power JB, Cocking EC, Bennett MD, et al. Molecular systematics of *Solanum* section *Lycopersicum* (*Lycopersicon*) using the nuclear ITS rDNA region. Theor Appl Genet. 2001;103(8):1216-22. doi: 10.1007/s001220100671.

5. Rick CM. Biosystematic studies in *Lycopersicon* and closely related species of *Solanum*. In: Hawkes JG, Lester RN, Skelding AD, editors. The Biology and Taxonomy of *Solanaceae*. New York: Academic Press; 1979. p. 667–77.

6. Dobin A, Davis CA, Schlesinger F, Drenkow J, Zaleski C, Jha S, et al. STAR: ultrafast universal RNA-seq aligner. Bioinformatics. 2013;29(1):15–21. doi: 10.1093/bioinformatics/bts635.

7. Li H, Handsaker B, Wysoker A, Fennell T, Ruan J, Homer N, et al. The Sequence Alignment/Map format and SAMtools. Bioinformatics. 2009;25(16):2078–9. doi: 10.1093/bioinformatics/btp352.

8. Pease JB, Rosenzweig BK. Encoding data using biological principles: the Multisample Variant Format for phylogenomics and population genomics. Trans Comp Biol Bioinformat. 2015; (In Press.).

9. Grabherr MG, Haas BJ, Yassour M, Levin JZ, Thompson DA, Amit I, et al. Full-length transcriptome assembly from RNA-Seq data without a reference genome. Nat Biotechnol. 2011;29(7):644-52. doi: 10.1038/nbt.1883. PubMed Central PMCID: PMC3571712.

10. Wu TD, Watanabe CK. GMAP: a genomic mapping and alignment program for mRNA and EST sequences. Bioinformatics. 2005;21(9):1859-75. doi: 10.1093/bioinformatics/bti310.

11. Stamatakis A. RAxML version 8: A tool for phylogenetic analysis and post-analysis of large phylogenies. Bioinformatics. 2014;30(9):1312–3. doi: 10.1093/bioinformatics/btu033.

12. Mirarab S, Reaz R, Bayzid MS, Zimmermann T, Swenson MS, Warnow T. ASTRAL: genome-scale coalescent-based species tree estimation. Bioinformatics. 2014;30(17):i541–i8. doi: 10.1093/bioinformatics/btu462.

13. Liu L, Yu L, Edwards S. A maximum pseudo-likelihood approach for estimating species trees under the coalescent model. BMC Evol Biol. 2010;10(1):302. doi: 10.1186/1471-2148-10-302.

14. Labate JA, Robertson LD, Strickler SR, Mueller LA. Genetic structure of the four wild tomato species in the *Solanum peruvianum* *s.l.* species complex. Genome. 2014;57(3):169–80. doi: 10.1139/gen-2014-0003.

15. Maddison WP, Maddison DR. Mesquite: a modular system for evolutionary analysis. Version 3.04 2010. Available from: <http://mesquiteproject.org>.

16. Aberer AJ, Krompass D, Stamatakis A. Pruning rogue taxa improves phylogenetic accuracy: an efficient algorithm and webservice. Syst Biol. 2013;62(1):162-6. doi: 10.1093/sysbio/sys078.

17. Maddison WP. Gene trees in species trees. Syst Biol. 1997;46(3):523-36. doi: 10.1093/sysbio/46.3.523.

18. Bouckaert RR. DensiTree: making sense of sets of phylogenetic trees. Bioinformatics. 2010;26(10):1372-3. doi: 10.1093/bioinformatics/btq110.

19. Paradis E, Claude J, Strimmer K. APE: Analyses of Phylogenetics and Evolution in R language. Bioinformatics. 2004;20(2):289-90. doi: 10.1093/bioinformatics/btg412.

20. Suh A, Smeds L, Ellegren H. The dynamics of incomplete lineage sorting across the ancient adaptive radiation of Neoavian birds. PLoS Biol. 2015;13(8):e1002224. doi: 10.1371/journal.pbio.1002224.

21. Yang Z. PAML 4: Phylogenetic Analysis by Maximum Likelihood. Mol Biol Evol. 2007;24(8):1586–91. doi: 10.1093/molbev/msm088.

22. Sanderson MJ. r8s: inferring absolute rates of molecular evolution and divergence times in the absence of a molecular clock. Bioinformatics. 2003;19(2):301–2. doi: 10.1093/bioinformatics/19.2.301.

23. Särkinen T, Bohs L, Olmstead R, Knapp S. A phylogenetic framework for evolutionary study of the nightshades (Solanaceae): a dated 1000-tip tree. BMC Evol Biol. 2013;13(1):214. doi: 10.1186/1471-2148-13-214.

24. The Tomato Genome Consortium. The tomato genome sequence provides insights into fleshy fruit evolution. Nature. 2012;485(7400):635–41. doi: 10.1038/nature11119.

25. Hudson RR. Generating samples under a Wright–Fisher neutral model of genetic variation. Bioinformatics. 2002;18(2):337–8. doi: 10.1093/bioinformatics/18.2.337.

26. Rodriguez F, Wu F, Ané C, Tanksley S, Spooner D. Do potatoes and tomatoes have a single evolutionary history, and what proportion of the genome supports this history? BMC Evol Biol. 2009;9(1):191. doi: 10.1186/1471-2148-9-191.

27. Peralta IE, Spooner DM. Granule-bound starch synthase (GBSSI) gene phylogeny of wild tomatoes (*Solanum* L. section *Lycopersicon* [Mill.] Wettst. subsection *Lycopersicon*). Am J Bot. 2001;88(10):1888-902.

28. Palmer JD, Zamir D. Chloroplast DNA evolution and phylogenetic relationships in *Lycopersicon*. Proc Natl Acad Sci USA. 1982;79(16):5006–10.

29. Spooner DM, Peralta IE, Knapp S. Comparison of AFLPs with other markers for phylogenetic inference in wild tomatoes [*Solanum* L. section *Lycopersicon* (Mill.) Wettst.]. Taxon. 2005;54(1):43-61.

30. Peralta IE, Spooner DM. Morphological characterization and relationships of wild tomatoes (*Solanum* L. Sect. *Lycopersicon*). Syst Bot Monogr. 2005;104:227.

31. Szinay D, Wijnker E, van den Berg R, Visser RGF, de Jong H, Bai Y. Chromosome evolution in *Solanum* traced by cross-species BAC-FISH. New Phytol. 2012;195(3):688–98. doi: 10.1111/j.1469-8137.2012.04195.x.

32. Anderson LK, Covey PA, Larsen LR, Bedinger P, Stack SM. Structural differences in chromosomes distinguish species in the tomato clade. Cytogenet Genome Res. 2010;129(1–3):24–34. doi: 10.1159/000313850.

33. Darwin SC, Knapp S, Peralta IE. Taxonomy of tomatoes in the Galápagos Islands: native and introduced species of *Solanum* section *Lycopersicon* (Solanaceae). Syst Biodivers. 2003;1(1):29-53. doi: 10.1017/S1477200003001026.

34. Rick CM, Fobes JF, Holle M. Genetic variation in *Lycopersicon pimpinellifolium*: Evidence of evolutionary change in mating systems. Pl Syst Evol. 1977;127(2-3):139-70. doi: 10.1007/BF00984147.

35. Zuriaga E, Blanca JM, Cordero L, Sifres A, Blas-Cerdán WG, Morales R, et al. Genetic and bioclimatic variation in *Solanum pimpinellifolium*. Genet Resour Crop Evol. 2009;56(1):39-51. doi: 10.1007/s10722-008-9340-z.

36. Nakazato T, Housworth EA. Spatial genetics of wild tomato species reveals roles of the Andean geography on demographic history. Am J Bot. 2011;98(1):88-98. doi: 10.3732/ajb.1000272.

37. Caicedo AL. Geographic diversity cline of R gene homologs in wild populations of *Solanum pimpinellifolium* (Solanaceae). Am J Bot. 2008;95(3):393-8. doi: 10.3732/ajb.95.3.393.

38. Koenig D, Jiménez-Gómez JM, Kimura S, Fulop D, Chitwood DH, Headland LR, et al. Comparative transcriptomics reveals patterns of selection in domesticated and wild tomato. Proc Natl Acad Sci USA. 2013;110(28):E2655-E62. doi: 10.1073/pnas.1309606110.

39. Labate J, Robertson L. Evidence of cryptic introgression in tomato (*Solanum lycopersicum* L.) based on wild tomato species alleles. BMC Plant Biol. 2012;12(1):133. doi: 10.1186/1471-2229-12-133.

40. Blanca J, Cañizares J, Cordero L, Pascual L, Diez MJ, Nuez F. Variation revealed by SNP genotyping and morphology provides insight into the origin of the tomato. PLoS One. 2012;7(10):e48198. doi: 10.1371/journal.pone.0048198.

41. Lucatti A, van Heusden A, de Vos R, Visser R, Vosman B. Differences in insect resistance between tomato species endemic to the Galapagos Islands. BMC Evol Biol. 2013;13(1):175. doi: 10.1186/1471-2148-13-175.

42. Baek YS, Covey PA, Petersen JJ, Chetelat RT, McClure B, Bedinger PA. Testing the SI × SC rule: pollen–pistil interactions in interspecific crosses between members of the tomato clade (*Solanum* section *Lycopersicon*, Solanaceae). Am J Bot. 2015;102(2):302–11

doi: 10.3732/ajb.1400484.

43. Moyle LC. Ecological and evolutionary genomics in the wild tomatoes (*Solanum* sect. *Lycopersicon*). Evolution. 2008;62(12):2995–3013. doi: 10.1111/j.1558-5646.2008.00487.x.

44. Vosters SL, Jewell CP, Sherman NA, Einterz F, Blackman BK, Moyle LC. The timing of molecular and morphological changes underlying reproductive transitions in wild tomatoes (*Solanum* sect. *Lycopersicon*). Mol Ecol. 2014;23(8):1965–78. doi: 10.1111/mec.12708.

45. Peralta IE, Knapp SK, Spooner DM. New species of wild tomatoes (*Solanum* section *Lycopersicon*: Solanaceae) from Northern Peru. Syst Bot. 2005;30(2):424–34. doi: 10.1600/0363644054223657.

46. Nakazato T, Franklin RA, Kirk BC, Housworth EA. Population structure, demographic history, and evolutionary patterns of a green-fruited tomato, *Solanum peruvianum* (Solanaceae), revealed by spatial genetics analyses. Am J Bot. 2012;99(7):1207-16. doi: 10.3732/ajb.1100210.

47. Nakazato T, Warren DL, Moyle LC. Ecological and geographic modes of species divergence in wild tomatoes. Am J Bot. 2010;97(4):680–93. doi: 10.3732/ajb.0900216.

48. Miller J, Kostyun J. Functional gametophytic self-incompatibility in a peripheral population of *Solanum peruvianum* (Solanaceae). Heredity. 2011;107(1):30-9. doi: 10.1038/hdy.2010.151.

49. Hartley AJ, Chong G, Houston J, Mather AE. 150 million years of climatic stability: evidence from the Atacama Desert, northern Chile. J Geol Soc London. 2005;162(3):421–4. doi: 10.1144/0016-764904-071.

50. Rech JA, Currie BS, Michalski G, Cowan AM. Neogene climate change and uplift in the Atacama Desert, Chile. Geology. 2006;34(9):761-4. doi: 10.1130/g22444.1.

51. Rick CM, Tanksley SD. Genetic variation in *Solanum pennellii*: Comparisons with two other sympatric tomato species. Pl Syst Evol. 1981;139(1-2):11-45. doi: 10.1007/BF00983920.

52. Sifres A, Blanca J, Nuez F. Pattern of genetic variability of *Solanum habrochaites* in its natural area of distribution. Genet Resour Crop Evol. 2011;58(3):347-60. doi: 10.1007/s10722-010-9578-0.

53. Picard D, Sempere T, Plantard O. Direction and timing of uplift propagation in the Peruvian Andes deduced from molecular phylogenetics of highland biotaxa. Earth Planet Sci Lett. 2008;271(1–4):326–36. doi: 10.1016/j.epsl.2008.04.024.

54. Doan TM. A south‐to‐north biogeographic hypothesis for Andean speciation: evidence from the lizard genus *Proctoporus* (Reptilia, Gymnophthalmidae). J Biogeogr. 2003;30(3):361–74. doi: 10.1046/j.1365-2699.2003.00833.x.

55. Hughes C, Eastwood R. Island radiation on a continental scale: exceptional rates of plant diversification after uplift of the Andes. Proc Natl Acad Sci USA. 2006;103(27):10334-9. doi: 10.1073/pnas.0601928103.

56. Chaves JA, Weir JT, Smith TB. Diversification in *Adelomyia* hummingbirds follows Andean uplift. Mol Ecol. 2011;20(21):4564-76. doi: 10.1111/j.1365-294X.2011.05304.x.

57. Luebert F, Weigend M. Phylogenetic insights into Andean plant diversification. Front Ecol Evol. 2014;2:27. doi: 10.3389/fevo.2014.00027.

58. Dillon MO, Tu T, Xie L, Quipuscoa Silvestre V, Wen J. Biogeographic diversification in *Nolana* (Solanaceae), a ubiquitous member of the Atacama and Peruvian Deserts along the western coast of South America. J Syst Evol. 2009;47(5):457–76. doi: 10.1111/j.1759-6831.2009.00040.x.

59. Garzione CN, Hoke GD, Libarkin JC, Withers S, MacFadden B, Eiler J, et al. Rise of the Andes. Science. 2008;320(5881):1304–7. doi: 10.1126/science.1148615.

60. Baker PA, Fritz SC, Dick CW, Eckert AJ, Horton BK, Manzoni S, et al. The emerging field of geogenomics: constraining geological problems with genetic data. Earth-Sci Rev. 2014;135(0):38-47. doi: 10.1016/j.earscirev.2014.04.001.

61. Garzione CN, Auerbach DJ, Jin-Sook Smith J, Rosario JJ, Passey BH, Jordan TE, et al. Clumped isotope evidence for diachronous surface cooling of the Altiplano and pulsed surface uplift of the Central Andes. Earth Planet Sci Lett. 2014;393(0):173–81. doi: 10.1016/j.epsl.2014.02.029.

62. Garreaud RD, Molina A, Farias M. Andean uplift, ocean cooling and Atacama hyperaridity: a climate modeling perspective. Earth Planet Sci Lett. 2010;292(1–2):39–50. doi: 10.1016/j.epsl.2010.01.017.

63. Edwards EJ, Osborne CP, Strömberg CA, Smith SA. The origins of C_4_ grasslands: integrating evolutionary and ecosystem science. Science. 2010;328(5978):587-91. doi: 10.1126/science.1177216.

64. Särkinen T, Pennington RT, Lavin M, Simon MF, Hughes CE. Evolutionary islands in the Andes: persistence and isolation explain high endemism in Andean dry tropical forests. J Biogeogr. 2012;39(5):884-900. doi: 10.1111/j.1365-2699.2011.02644.x.
