# Supplementary material for: Phylogenomics Reveals Three Sources of Adaptive Variation during a Rapid Radiation
Source: PLoS Biol. 2016 Feb 12;14(2):e1002379. doi: 10.1371/journal.pbio.1002379 (PMC4752443; doi:10.1371/journal.pbio.1002379)
Supplement: S2 Text — (DOCX) [file pbio.1002379.s011.docx]

**S2 Text: Abstract en Español (Abstract in Spanish)**

Los eventos de especiación ocurren a menudo como estallidos rápidos de diversificación, pero los factores ecológicos y genéticos que promueven la radiación de especies continúan siendo materia de debate. En este estudio, usamos transcriptomas completos de las 13 especies de tomates silvestres (*Solanum* sect. *Lycoperiscon*), las cuales tienen mucha diversidad ecológica y reproductiva, para inferir la filogenia de las especies y los patrones de diversidad genética en este grupo. A pesar de la discordancia filogenética generalizada debido a la clasificación de variación ancestral (i.e., clasificación incompleta del linaje), estimamos que el origen de esta radiación ocurrió hace aproximadamente 2,5 millones de años. Asimismo, encontramos evidencia de al menos tres fuentes de variación genética adaptativa que estimulan la diversificación de las especies. En primer lugar, detectamos tanto introgresión histórica entre linajes ancestrales, así como introgresión reciente entre las poblaciones individuales en loci específicos cuyas funciones probablemente incluyen beneficios adaptativos. En segundo lugar, encontramos evidencia de evolución *de novo* para muchos genes, incluyendo loci involucrados en la producción del color rojo en los frutos. Por último, utilizando un método de “PhyloGWAS” (Estudio filogenético de asociación del genoma completo), detectamos alelos con clasificación incompleta asociados a ambientes específicos que provienen de diferentes especies pero que comparten condiciones ambientales similares. Tomando en cuenta todo del clado, inferimos que fracciones pequeñas pero importantes de la eucromatina en el genoma podrían contribuir por igual, aproximadamente, a cada una de estas tres fuentes de variación genética adaptativa. Estos resultados indican que diferentes fuentes de variación genética pueden promover la rápida diversificación y especiación en respuesta a nuevas oportunidades ecológicas. Nuestros resultados además concuerdan con nuevas perspectivas basadas en estudios de filogenómica, acerca de la complejidad de las radiaciones evolutivas antiguas y recientes.
